# Optimizing yeast for high-level production of kaempferol and quercetin

**DOI:** 10.1186/s12934-023-02084-4

**Published:** 2023-04-20

**Authors:** Musa Tartik, Juan Liu, Marta Tous Mohedano, Jiwei Mao, Yun Chen

**Affiliations:** 1 Department of Molecular Biology and Genetics, Faculty of Arts and Sciences, Bingol University, Bingol, 12000 Turkey; 2 Department of Life Sciences, Chalmers University of Technology, Kemivägen 10, Gothenburg, SE-412 96 Sweden

**Keywords:** Yeast, Flavonoids, Kaempferol, Quercetin

## Abstract

**Background:**

Two important flavonoids, kaempferol and quercetin possess remarkably potent biological impacts on human health. However, their structural complexity and low abundance in nature make both bulk chemical synthesis and extraction from native plants difficult. Therefore microbial production via heterologous expression of plant enzymes can be a safe and sustainable route for their production. Despite several attempts reported in microbial hosts, the production levels of kaempferol and quercetin still stay far behind compared to many other microbial-produced flavonoids.

**Results:**

In this study, *Saccharomyces cerevisiae* was engineered for high production of kaempferol and quercetin in minimal media from glucose. First, the kaempferol biosynthetic pathway was reconstructed via screening various F3H and FLS enzymes. In addition, we demonstrated that amplification of the rate-limiting enzyme AtFLS could reduce the dihydrokaempferol accumulation and improve kaempferol production. Increasing the availability of precursor malonyl-CoA further improved the production of kaempferol and quercetin. Furthermore, the highest amount of 956 mg L^− 1^ of kaempferol and 930 mg L^− 1^ of quercetin in yeast was reached in fed-batch fermentations.

**Conclusions:**

*De novo* biosynthesis of kaempferol and quercetin in yeast was improved through increasing the upstream naringenin biosynthesis and debugging the flux-limiting enzymes together with fed-batch fermentations, up to gram per liter level. Our work provides a promising platform for sustainable and scalable production of kaempferol, quercetin and compounds derived thereof.

**Supplementary Information:**

The online version contains supplementary material available at 10.1186/s12934-023-02084-4.

## Introduction

Flavonoids are a broad group of polyphenolic secondary metabolites present in plants to execute essential functions such as cell signaling, UV protection, attenuation of oxidative injury, and prevention of microbial infections [1–3]. They can be classified into several sub-groups including more than 9000 derivates owing to their chemical skeleton structured by two aromatic rings connected by a heterocyclic pyran C ring, which can be modified with the addition of various functional groups [4, 5]. Diverse *in vitro* and *in vivo* analysis indicated that the bioactive properties of flavonoids are not just limited to plants but can also be used to afford significant benefits in human health problems such as cancer, cardiovascular troubles, and ageing [3, 6–8]. Two important flavonoids, kaempferol and quercetin, are of particular interest for their remarkable impacts on human health [9–14]. They both possess potent biological activities: causing bacterial death by disrupting membrane potential [1, 15, 16], acting directly as free-radical scavengers preventing cellular damages [17], binding diverse pro-inflammatory and pro-carcinogenic agents for detoxification [18, 19], contributing antitumor activity by inhibiting cyclins and increasing tumor suppressor protein concentration [20], and blocking angiogenesis with VEGF inhibition [21]. However, their structural complexity and low abundance in nature hamper both bulk chemical synthesis and extraction from native plants. Microbial production via heterologous expression of plant enzymes can be a safe and sustainable route for production of these high-value compounds.

**Fig. 1:**
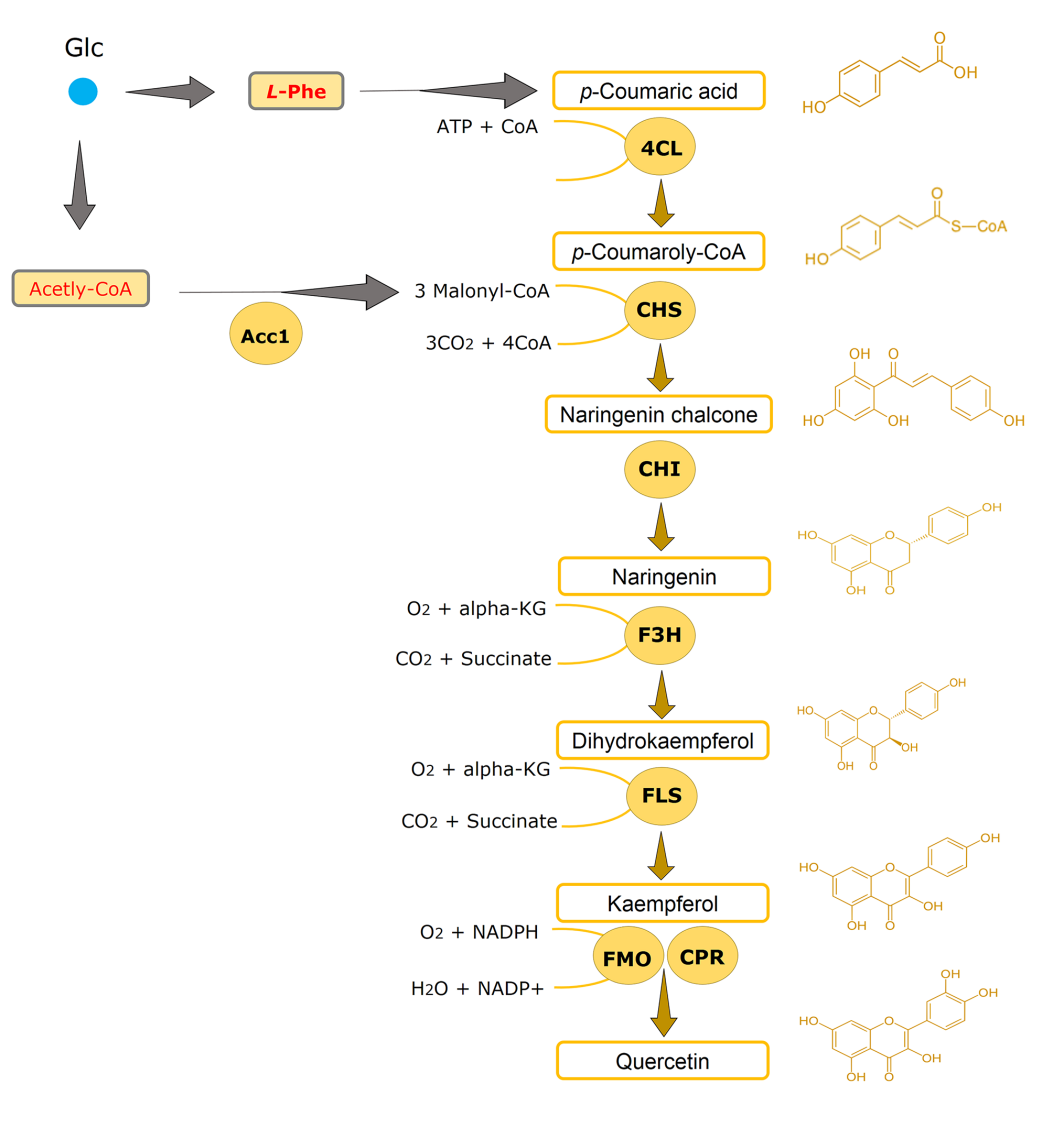
Production of flavonoids kaempferol and quercetin in *S. cerevisiae* directly from glucose. _L_-Phenlalanine (_L_-Phe) derived from the shikimate and aromatic amino acid biosynthesis pathway is a precursor for *p*-coumaric acid formation. *p-*Coumaric acid is further catalyzed by a *p*-coumaric acid: CoA ligase (4CL), a chalcone synthase (CHS), a chalcone isomerase (CHI) to produce naringenin, which is an important junction to divaricate to different flavonoids. Naringenin can be further converted by a flavanone 3-hydroxylase (F3H) and flavonol synthase (FLS) to generate kaempferol, followed by a flavonoid monooxygenase (FMO) together with a cytochrome P450 reductase (CPR) to finaly give quercetin. Glc, glucose; alpha-KG, alpha-ketoglutarate

The biosynthesis pathway of flavonoids has been well studied and made it readily applicable in heterologous microbial cell factories [2]. It starts with the conversion of _L_-tyrosine or _L_-phenylalanine to *p*-coumaric acid, followed by catalysis via a *p*-coumaric acid: CoA ligase (4CL), a chalcone synthase (CHS), a chalcone isomerase (CHI) to form naringenin (Fig. 1). Naringenin is an important central intermediate to produce diverse phenylpropanoids, including kaempferol and quercetin. Specifically, flavanone 3-hydroxylase (F3H) and flavonol synthase (FLS) are two consecutive enzymes required to convert naringenin to kaempferol through an intermediate dihydrokaempferol (Fig. 1). Following, quercetin is produced downstream from kaempferol by a flavonoid monooxygenase (FMO) together with a cytochrome P450 reductase (CPR).

Not surprisingly, production of kaempferol and quercetin have been reported in several microbial hosts either via supplementation of intermediates or directly from a carbon source, such as *Escherichia coli* [22], *Corynebacterium glutamicum* [23], *Streptomyces* [24] and yeast *Saccharomyces cerevisiae* [25–28]. The introduction of the two heterologous genes F3H and FLS into *E.coli* and yeast has enabled the production of kaempferol with the supplement of precursor naringenin [22, 25]. To avoid adding expensive precursor metabolites, *de novo* production of kaempferol has been developed in yeast. With the advantage of a high coumaric acid-producing strain, Rodriguez et al., established *de novo* production of kaempferol from glucose [26]. However, the production of kaempferol only reached 26 mg L^− 1^. Reasoning the insufficient supply of precursor malonyl-CoA, the acetyl-CoA biosynthetic pathway was overexpressed together with *p*-coumaric acid supplementation resulting in 18.76 mg L^− 1^ of kaempferol in yeast, further up to 66.29 mg L^− 1^ by fed-batch fermentations [28]. In a more recent study, optimization of the kaempferol biosynthesis pathway and blocking the phenylethanol biosynthesis pathway have increased the production of kaempferol to 86 mg L^− 1^ [27]. Similarly, the highest quercetin production was only 20.4 mg L^− 1^ in a kaempferol producing yeast strain [26]. However, compared to many other microbial produced flavonoids, for instance, up to gram per liter of naringenin has been achieved in *S. cerevisiae* [29–31] and *Yarrowia lypolytica* [32], both kaempferol and quercetin levels still stay far behind.

In this study, we aim to improve the *de novo* biosynthesis of kaempferol and quercetin in yeast. Through increasing the upstream naringenin biosynthesis, debugging the flux-limiting enzymes together with fed batch fermentations, both kaempferol and quercetin production reached up to gram per liter level.

## Materials and methods

### Strains and reagents

All plasmids (Supplementary Data 1 - Table 1) were constructed and amplified with *E. coli* DH5α. The list of used *S. cerevisiae* strains is available in Supplementary Data 1 - Table 2. DNA polymerase kits were purchased: High-fidelity Phusion from New England Biolabs, PrimeStar DNA polymerase, and SapphireAmp® Fast PCR Master Mix from TaKaRa Bio. All oligonucleotides (Supplementary Data 1 - Table 4) were synthesized at IDT Biotechnology. All other chemicals were purchased from Sigma-Aldrich.

All codon-optimized heterologous genes were synthesized from GenScript and are listed in Supplementary Data 1 - Table 3.

### Strain cultivation

Competent yeast cells were prepared by cultivation in YPD media including 20 g L^− 1^ glucose (VWR), 10 g L^− 1^ yeast extract (Merck Millipore) and 20 g L^− 1^ peptone (Thermo Fisher Scientific). To select yeast transformants containing *URA3* marker-based plasmids, synthetic complete medium without uracil (SC-URA) was used, consisting of 20 g L^− 1^ agar (Merck Millipore), 20 g L^− 1^ glucose, 6.7 g L^− 1^ yeast nitrogen base (YNB) without amino acids (FormediumTM,) and 0.77 g L^− 1^ complete supplement mixture lacking uracil (CSM-URA). The selective SC with 5-fluoroorotic acid (SC + 5-FOA) media consisting of 0.8 g L^− 1^ 5-FOA, 0.77 g L^− 1^ complete supplement mixture and 6.7 g L^− 1^ YNB, was used to lose the URA3 marker-based plasmids.

For the production of kaempferol and quercetin, five single colonies with relevant heterologous pathways were selected and cultivated in 14 mL falcon tubes with 1.5 mL of minimal media consisting of 7.5 g L^− 1^ (NH_4_)_2_SO_4_, 14.4 g L^− 1^ KH_2_PO_4_, 0.5 g L^− 1^ MgSO_4_·7H_2_O, 30 g L^− 1^ glucose, 2 mL L^− 1^ trace metals solution (3.0 g L^− 1^ FeSO_4_·7H_2_O, 4.5 g L^− 1^ ZnSO_4_·7H_2_O, 4.5 g L^− 1^ CaCl_2_·2H_2_O, 0.84 g L^− 1^ MnCl_2_·2H_2_O, 0.3 g L^− 1^ CoCl_2_·6H_2_O, 0.3 g L^− 1^ CuSO_4_·5H_2_O, 0.4 g L^− 1^ Na_2_MoO_4_·2H_2_O, 1.0 g L^− 1^ H_3_BO_3_, 0.1 g L^− 1^ KI, and 19.0 g L^− 1^ Na_2_EDTA·2H_2_O) and 1 mL L^− 1^ vitamin mix solution (0.05 g L^− 1^ D-biotin, 1.0 g L^− 1^ D-pantothenic acid Hemi-calcium salt, 1.0 g L^− 1^ thiamin–HCl, 1.0 g L^− 1^ pyridoxine–HCl, 1.0 g L^− 1^ nicotinic acid, 0.2 g L^− 1^ 4 aminobenzoic acid, and 25.0 g L^− 1^ myo-inositol) [33]. Additionally, the medium was supplemented with 60 mg L^− 1^ uracil if required. Tubes were precultured at 30 °C with 200-rpm overnight. Then, they were re-inoculated in 125 mL non-baffled flasks including 20 mL of fresh minimal media at 0.05 of initial optical density (OD_600_) and were kept at 200 rpm, 30 °C for 72 h. For shake flask fed-batch mimicking fermentations, six tablets of FeedBeads [34] (SMFB08001, Kuhner Shaker) with slow releasing glucose were used instead of 30 g L^− 1^ glucose as the sole carbon source and cultivated for 96 h at 30 °C with 200-rpm agitation.

The fed-batch cultivation was done in quadruplicates (for strain MTK43A) and in triplicates (for strain MTQ13A) using a 1 L parallel bioreactor system (DasGip Parallel Bioreactor System, DasGip, Germany). The used minimal medium of the starting cultured consisted of 30 g L^− 1^ glucose, 15 g L^− 1^ (NH_4_)_2_SO_4_, 9 g L^− 1^ KH_2_PO_4_, 1.5 g L^− 1^ MgSO_4_•7H_2_O, 180 mg L^− 1^ uracil, 3 mL L^− 1^ trace metal solution and 3 mL L^− 1^ vitamin solution. Initially, the reactor had 250 mL of minimal media adjusted at pH 5.6 which was controlled by the addition of 2 M HCl and 4 M KOH. The agitation (set at 500 rpm), the pH and the temperature (set at 30 °C) were controlled using the DasGip Control 4.0 System. The aeration was initially set at 30 L h^− 1^ and was controlled by a DasGip MX4/4 module. The dissolved oxygen in the media was maintained above 30%. However, when the dissolved oxygen would decrease below 30% the aeration would increase to 48 L/h and the agitation to 900 rpm. The addition of acid, base and high concentrated glucose feeding was performed by DasGip MP8 multi-pump modules (pump head tubing: 0.5 mm ID, 1.0 mm wall thickness). The composition of the off-gas was monitored using a DasGip Off Gas Analyser GA4. The feed was started by an exponentially feeding rate according to Supplementary Data 2 after the glucose and ethanol were consumed. The fermenters for kaempferol production were clogged during the feeding process, the feeding was shifted back 24 h at 48 h after feeding started. The feeding media contained 200 g L^− 1^ glucose, 15 g L^− 1^ (NH_4_)_2_SO_4_, 9 g L^− 1^ KH_2_PO_4_, 1.5 g L^− 1^ MgSO_4_•7H_2_O, 180 mg L^− 1^ uracil, 3 mL L^− 1^ trace metal solution and 3 mL L^− 1^ vitamin solution. Furthermore, 0.25 mL of antifoam was added to each reactor to prevent foaming.

All bacterial cultivation was performed in a Luria-Bertani (LB) medium consisting of 10 g L^− 1^ NaCI (Merck Millipore), 5 g L^− 1^ yeast extract (Merck Millipore) and 10 g L^− 1^ peptone from casein (tryptone) (Thermo Fisher Scientific). The liquid and solid selective media were supplemented with ampicillin.

### Genetic modifications

All genetic manipulations were performed in the background of IMX581 (MATa ura3-52 can1Δ::cas9-natNT2 *TRP1 LEU2 HIS3*) with CRISPR-Cas9 technology [35]. All native promoters, terminators, and genes were amplified from the genomic DNA of IMX581. All required DNA fragments of expression modules were amplified with relevant primer pairs (Supplementary Data 1 - Table 4). High-fidelity Phusion DNA polymerase was usually used to amplify single DNA fragments while PrimeStar HS polymerase was selected to fuse DNA parts for generating integration modules. The entire cloning procedure to construct all functional integration modules was performed according to the overlapping extension PCR (OE-PCR) protocol [36], and all used integration modules are listed in Supplementary Data 1 - Table 5.

All constructed modules were integrated into the targeted genomic loci enabling stable and high-level expression of heterologous genes [37]. To clearly explain module construction, herein we define the M1 cassette in detail as an example in particular, which was designed to integrate XI-3 locus in strain IMX581. M1 module (XI-3 us-TEF1p-OsF3H-PRM1t-TDH2t-AtFLS-TPI1p- XI-3 ds) includes two heterologous genes (OsF3H and AtFLS) and native promoters (*TEF1p* and *TPI1p*), terminators (*PRM1t* and *TDH2t*), XI-3 loci (the upstream and downstream of gRNA sequence cut by Cas9). The relevant primer pairs having the residue sequence of front or back DNA part in the constructed module to overlap were used to amplify promoters (P3/P4 for *TEF1p* and P27/P28 for *TPI1p*), terminators (P11/P12 for *PRM1t* and P15/P16 for *TDH2t*), and XI-3 us (P1/P2) and ds (P33/P34) arms (Supplementary Data 1 - Table 6) from IMX581 genomic DNA. Both OsF3H (P7/P8) and AtFLS (P21/P22) heterologous genes were amplified from their backbone plasmids (Supplementary Data 1 - Table 1) with specific primers. All DNA parts were overlapped with PCR to construct the entire module without any primers. Then, yeast strain IMX581 was co-transformed with purified M1 module (50–100 ng/kb) and gRNA plasmid pQC006 (~ 300–500 ng) according to the stated protocols [35, 38] and cultivated on SC-URA plates to select desired transformants. Picked colonies were verified with SapphireAmp® Fast PCR Master Mix. Subsequently, five colonies having the correct heterologous DNA module were inoculated in YPD for overnight cultivation. Then each of them streaked onto SC + FOA plates to drop off gRNA plasmid and recycle the URA3 marker which can be used for further genetic modifications.

The specific guide RNA was selected among all potential candidates for genomic loci (X-2, XI-2, XI-3, and XI-5) to avoid any possible off-target in the whole CEN. PK113-7D genome with CRISPRdirect tool [39] which is access-free at http://crispr.dbcls.jp/. The gRNA vectors were previously constructed in our research group by the Gibson assembly method protocol [40].

In addition, a mutant version of endogenous Acc1^S659A,S1157A^ [41] has been integrated into the yeast genome in chromosome V (V::TPIp-Acc1^S659A,S1157A^-TDH2t) to increase the malonyl-CoA flux towards the flavonoid production pathway. The Acc1^S659A,S1157A^ gene has been amplified from the pAD vector previously constructed in the group [41].

### Extraction and quantification of flavonoids

High-performance liquid chromatography (HPLC) was used to determine and quantify metabolites produced in whole-yeast cultures as described before [26]. Briefly, 0.5 mL of absolute ethanol (100% v/v) was thoroughly mixed with an equal volume of yeast culture cultivated either in the shake-flask batch for 72 h or with FeedBeads for 96 h and centrifuged at 13,500 × g for 5 min. Additionally, samples can be diluted with different absolute ethanol volumes. The supernatants were analyzed with Dionex Ultimate 3000 HPLC (Thermo Fisher Scientific) having a photodiode array detector. Metabolites from 10 µL of samples were separated in Discovery HS F5 150- mm × 4.6-mm column (particle size 5 μm) (Sigma-Aldrich) kept at 30 °C.

Two solvents were used for HPLC analysis: 10 mM ammonium formate pH 3 (adjusted with formic acid) (A) and acetonitrile (B). Dihydrokaempferol, naringenin, quercetin, and kaempferol were detected with a gradient method: a constant flow rate (1.2 mL min^− 1^), the initial combination was 15% of solvent B (0–1.5 min), the gradient was increased from 15 to 20% (1.5–3 min), then there is another ramp from 20 to 45% (3–24 min), after that a quick decrease from 45 to 15% (24–25 min) and maintained at 15% for 1.5 min (25–26.5 min). The compounds were detected: dihydrokaempferol at 7.70 min (280 nm), naringenin at 14.21 min (280 nm), kaempferol at 14.9 min (370 nm) and quercetin at 11.2 min (370 nm).

## Results and discussion

### Optimizing the biosynthesis of kaempferol from naringenin

**Fig. 2:**
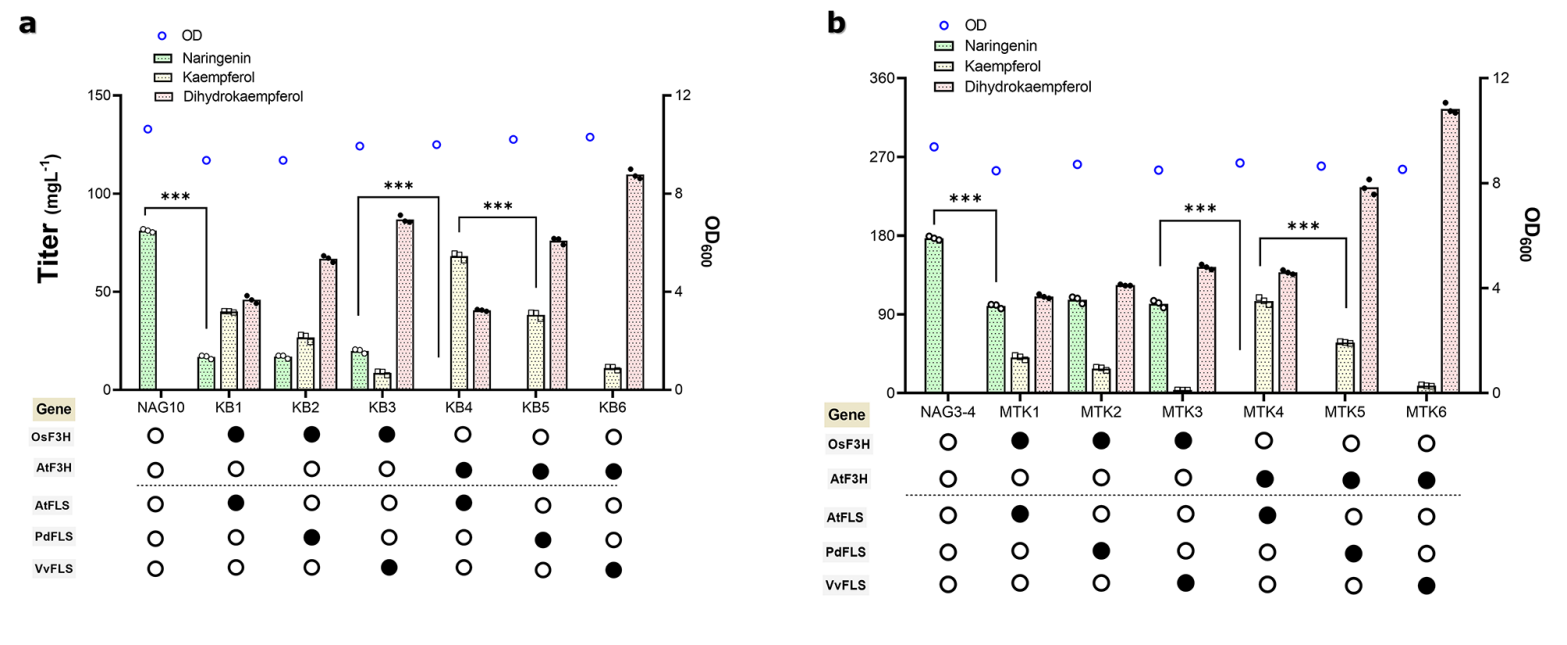
Production of kaempferol from different naringenin platform strains. (**a**) Screening a variety of F3H and FLS from different plant sources to determine the best combination by testing in NAG10 strain, being capable to produce 80 mg L^− 1^ of naringenin. (**b**) Investigating the same enzyme combinations in the background of NAG3-4 strain, which has a higher naringenin production (180 mg L^−^1). AtF3H from *Arabidopsis thaliana*, OsF3H from *Oryza sativa*, AtFLS from *A. thaliana*, PdFLS from *Populus deltoides*, and VvFLS from *Vitis vinifera*. The black circle shows the existence of the gene in the strain, instead of the white circle representing its absence. Cells were cultured in a defined minimal medium in shake flask conditions, and samples were taken after 72 h of growth for naringenin, dihydrokaempferol, and kaempferol detection. Statistical analysis was performed by using one-way ANOVA, Tukey’s multiple comparison test * < 0.05, ** < 0.01, *** < 0.001). All data represent the mean ± standard deviation of 3 biologically independent samples

For *de novo* production of kaempferol in *S. cerevisiae*, its biosynthetic pathway was reconstituted in a previously constructed naringenin producing strain NAG10 [31]. The naringenin producer contains a _L_-phenylalanine-based pathway including one copy of each gene from phenylalanine to naringenin integrated into the genome, in addition to feedback resistant variants of ARO4* and ARO7*, and extra copies of *ARO1/2/3* together with *Ec*aroL from *E. coli*, which has been previously proved to significantly improve *p*-coumaric acid production [42]. Since naringenin branches out to kaempferol via two catalytic steps by two consecutive enzymes (Fig. 1), F3H and FLS, various combinations of F3H and FLS from different origins (F3H from *Arabidopsis thaliana* (AtF3H) and *Oryza sativa* (OsF3H); FLS from *A. thaliana* (AtFLS), *Populus deltoides* (PdFLS), and *Vitis vinifera* (VvFLS)) were compared in parallel to point out the optimal candidates for kaempferol production. Different combinations resulted in strains KB1-KB6 (Fig. 2a).

While the starting strain NAG10 produced approximately 80 mg L^− 1^ of naringenin directly from glucose, all KB strains produced a different level of kaempferol, at a range of 8.6–68.2 mg L^− 1^ (Fig. 2a). At the same time, all KB strains accumulated different amounts of the intermediate dihydrokaempferol (40.1–109.7 mg L^− 1^) (Fig. 2a). Interestingly, KB1-KB3 strains all with OsF3H displayed naringenin accumulation whereas KB4-KB6 all with AtF3H had no naringenin left, indicating that OsF3H seems to have some limitations to convert its substrate naringenin compared to AtF3H. Comparing the amounts of dihydrokaempferol accumulated, there seemed to be a trend among FLS tested, VvFLS < PdFLS < AtFLS, regarding its efficiency of converting dihydrokaempferol into kaempferol. In addition, this trend was not depending on the F3H enzyme efficiency. These results on the efficiency of F3H and FLS contradicted with some previous studies. In one study AtF3H showed less efficiency compared with other F3H candidates [27] whereas in the other study AtF3H-PdFLS was the best combination for the conversion of naringenin into kaempferol [28], but AtF3H-AtFLS exhibited the highest production of kaempferol in our study. Such differences may well be caused by the differences in the strain background as well as in enzyme expressions such as promoters and terminators.

As shown in Fig. 2a, the best combination in KB4 strain containing AtF3H-AtFLS resulted in the highest kaempferol production at 68.2 mg L^− 1^. To be noted, this was achieved in shake flask cultivations with 30 g L^− 1^ glucose, while the titers were comparable to the highest titers reported to date by fed-batch fermentation or using rich medium [27, 28]. Compared to previous studies, our study was based on an optimized naringenin producing strain, indicating the importance of *in vivo* flux towards naringenin.

### Increasing naringenin supply improves kaempferol production

Next, we tested if increasing *in vivo* naringenin supply could improve kaempferol production. Therefore, we used a previously optimized naringenin producing strain, NAG3-4, in which the bottleneck downstream of *p*-coumaric acid was alleviated by increasing the copy number of downstream genes as *4CL:CHS:CHI* = 3:4:4 [31]. Indeed, this resulted in a titer of about 180 mg L^− 1^ naringenin being produced, more than doubled than that of NAG10. As heterologous enzyme function models are not clearly understood in microbial hosts, the introduced pathway may have different effects on metabolic flux and cell phenotypes in different strain backgrounds. Hence, different combinations of F3H and FLS was re-evaluated in NAG3-4 again, leading to MTK1-6 strains (Fig. 2b).

An almost similar trend was seen in MTK strains compared to KB strains in terms of naringenin and dihydrokaempferol accumulation. With AtF3H almost all naringenin was channeled to downstream products in MTK4-6 strains. However, when OsF3H was used more than 4 times of naringenin left in MTK1-3 strains compared to the corresponding KB01-03 strains (Fig. 2a and b), due to increased naringenin supply. Surprisingly, a significantly higher amount of dihydrokaempferol was formed in MTK6 strain, which deserves further investigation. Like the results in KB strains, AtFLS displayed the best activity to turn dihydrokaempferol into kaempferol among FLS candidates. The best kaempferol production was achieved in MTK4 strain with AtF3H-AtFLS, up to 105 mg L^− 1^ (Fig. 2b). However, AtFLS was still a bottleneck in kaempferol production as the presence of dihydrokaempferol in both KB and MTK backgrounds.

### Modulating the biosynthesis enzymes enhances kaempferol production

**Fig. 3:**
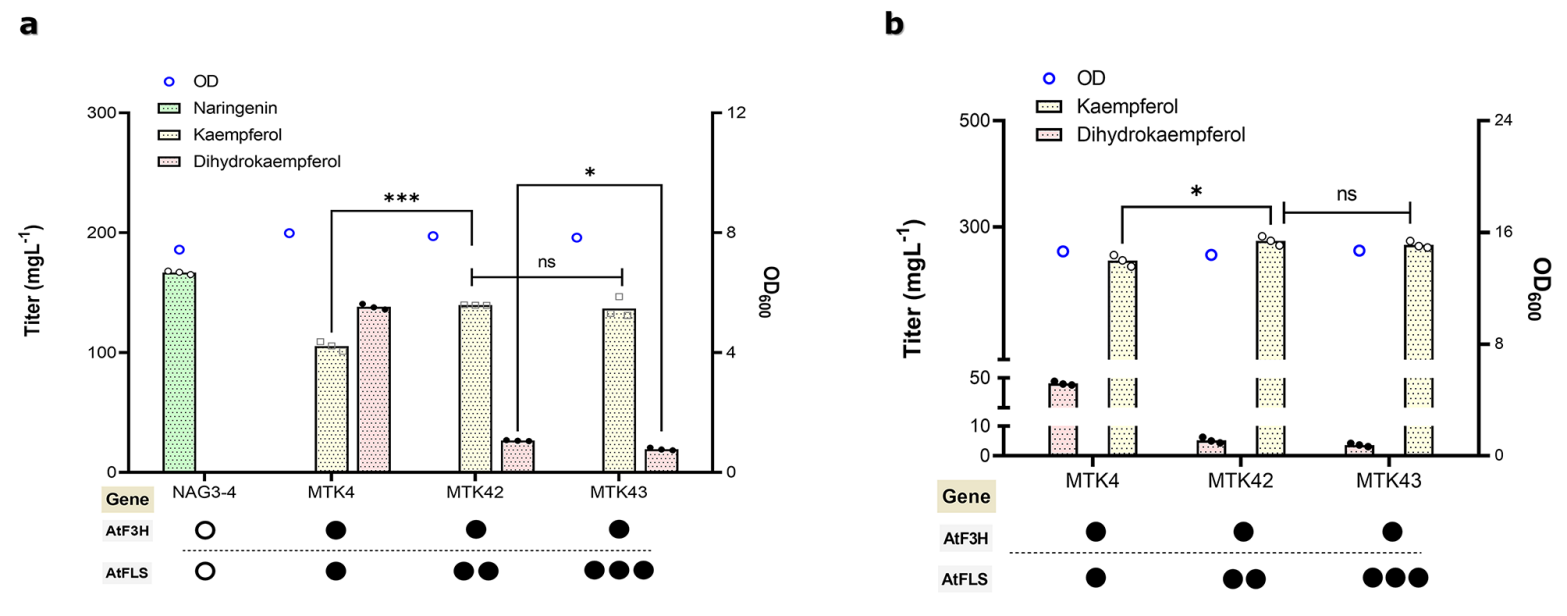
Optimization of the kaempferol biosynthetic pathway. (**a**) Comparison of kaempferol and dihydrokaempferol amount in yeast strains, which each has one, two, or three copies of AtFLS (FLS from *A. thaliana*). Cells were cultured in a defined minimal medium in shake flask conditions, and samples were taken after 72 h of growth for naringenin, dihydrokaempferol, and kaempferol detection. (**b**) Testing kaempferol producing strains in defined minimal medium in shake flask with six discs of FeedBeads, releasing glucose slowly and constantly. Samples were taken after 96 h of growth for naringenin, dihydrokaempferol, and kaempferol measurements. The black circle shows the existence of the gene in the strain, instead of the white circle representing its absence. Statistical analysis was performed by using one-way ANOVA, Tukey’s multiple comparison test (* < 0.05, ** < 0.01, *** < 0.001, ns, no significant difference). All data represent the mean ± standard deviation of 3 biologically independent samples

To alleviate the AtFLS bottleneck and further improve kaempferol production, we employed the simplest approach by increasing the copy number of AtFLS in MTK4. We hereby firstly introduced the second copy of AtFLS in MTK4, resulting in the MTK42 strain. This ended up with a significant decrease in dihydrokaempferol level from 136 mg L^− 1^ (MTK4) to 26 mg L^− 1^ (MTK42), simultaneously increased kaempferol production from 105 mg L^− 1^ to 139 mg L^− 1^ (Fig. 3a). But there still dihydrokaempferol remained in the culture, we therefore inserted the third copy of AtFLS in MTK42, forming MTK43. The impact of additional FLS was less pronounced, but it further reduced dihydrokaempferol level down to 19 mg L^− 1^ while remained the similar level of kaempferol. A further increase in the copy number of heterologous genes would be less efficient and may pose an extra burden on cell growth. Alternatively, gene screening from other plant species, or rational and/or evolutionary approaches to improve the activity of FLS would be considered in the future to eliminate dihydrokaempferol accumulation completely.

Furthermore, MTK4, MTK42, and MTK43 strains were tested under fed-batch mimicking conditions by using FeedBeads (Kühner AG, Germany), as it was shown to have higher flux in aromatic amino acid biosynthesis and in the supply of precursor *p*-coumaric acid [42]. The production of kaempferol in all strains almost doubled in comparison to the normal batch conditions (Fig. 3b). The best producer MTK43 reached 266 mg L^− 1^ kaempferol, with a low level of dihydrokaempferol (4 mg L^− 1^) compared to the background strain MTK4 (42 mg L^− 1^). This confirms that kaempferol production benefited from fed-batch mimicking conditions which prevent overflow metabolism via the slow release of glucose.

### Channeling kaempferol to quercetin production

Encouraged by high production of kaempferol in *S. cerevisiae*, we decided to further channel kaempferol to quercetin, which only requires one catalytic step but two enzymes, FMO and CPR (Fig. 1). A variety of FMO from different plant sources (FMO from *Glycine max* (GmFMO), *Arabidopsis thaliana* (AtFMO), and *Petunia hybrida* (PhFMO)) was screened to determine the best candidate. CPR has not been introduced again because the background strains have already a CPR from *A. thaliana* (AtATR2) together with an extra copy of CPR from *S. cerevisiae* (CYB5) [42]. Ultimately, the attempt ended up with MTQ1-3 strains and then they were tested in shake flask fermentations.

**Fig. 4:**
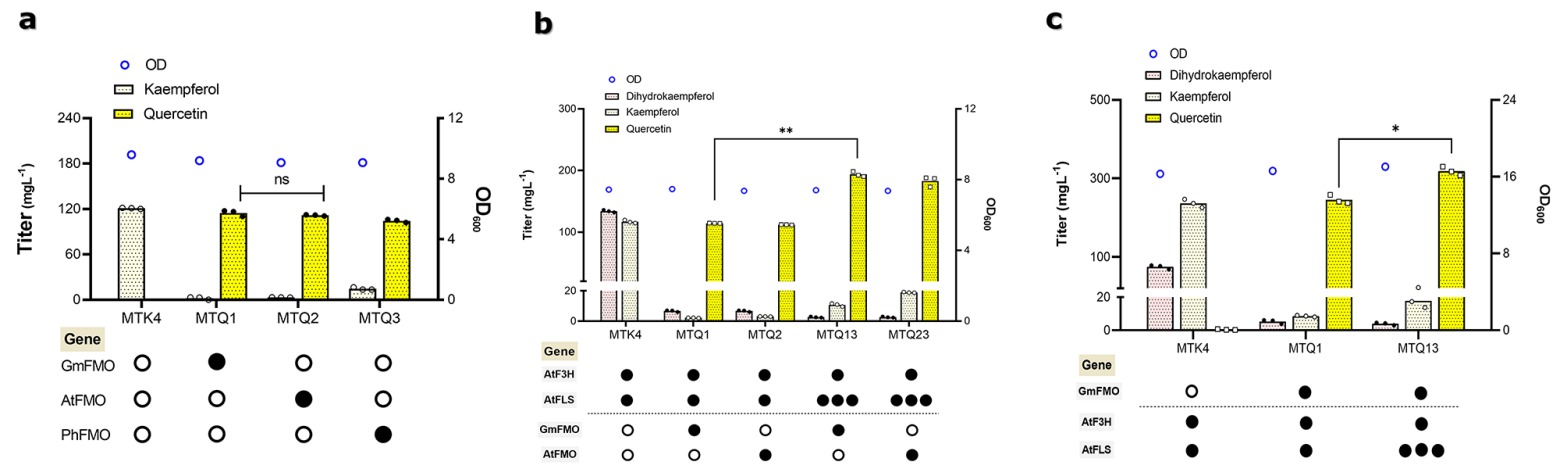
Extending the biosynthetic pathway from kaempferol to quercetin production. (**a**) Screening FMO from several plant sources to identify the best candidate by testing in MTK4 containing one copy of AtFLS. AtFMO from *A. thaliana*, GmFMO from *Glycine max*, and PhFMO from *Petunia hybrida*. CPR was not transferred to the strains because the background strains have already additional copy. (**b**) Effect of additional copies of AtFLS on quercetin production. The black circle shows the existence of the gene in the strain, instead of the white circle representing its absence. For (**a**) and (**b**), cells were cultured in a defined minimal medium in shake flask conditions, and samples were taken after 72 h of growth for quercetin, dihydrokaempferol, and kaempferol detection. **c** Assessing quercetin producing strains in defined minimal medium in shake flask with six discs of FeedBeads, releasing glucose slowly and constantly. Samples were taken after 96 h of growth for naringenin, dihydrokaempferol, and kaempferol measurements. Statistical analysis was performed by using one-way ANOVA, Tukey’s multiple comparison test (* < 0.05, ** < 0.01, *** < 0.001). All data represent the mean ± standard deviation of 3 biologically independent samples

Almost all FMO candidates showed a strong functionality to convert kaempferol to quercetin, besides PhFMO performed slightly worse than the other two as can be seen by the remaining kaempferol in MTQ3 (Fig. 4a). Surprisingly, when channeling the flux from kaempferol to quercetin, the accumulation of dihydrokaempferol was significantly reduced, less than 7 mg L^− 1^ in MTQ1 and MTQ2 (Fig. 4b), indicating the enzyme FMO was quite efficient to drain the flux towards quercetin. But dihydrokaempferol existed still in MTQ1 and MTQ2. We wondered if increase the expression of FLS could drain more flux to quercetin formation. Therefore, as before, two extra copies of AtFLS were introduced into MTQ1 and MTQ2, which results in MTQ13 and MTQ23. As expected, both strains had virtually no dihydrokaempferol accumulation. However, this led to kaempferol accumulation in both strains (10 mg L^− 1^ in MTQ13, 18 mg L^− 1^ in MTQ23), possibly due to the imbalance of FLS and FMO. Nevertheless, compared to MTQ1 and MTQ2, quercetin production increased by 69% and 63% in MTQ13 and MTQ23, respectively (Fig. 4b).

In addition, quercetin production was also evaluated under fed-batch mimicking conditions by using Feedbeads. Like kaempferol production, in comparison to batch conditions the quercetin titer was improved approximately by 2-fold in MTQ1 (245 mg L^− 1^) and 64% in MTQ13 (319 mg L^− 1^) (Fig. 4c), with small amounts of kaempferol and dihydrokaempferol left. Again, the results highlight the advantageous of slow release of glucose for improved quercetin production.

### High level production of kaempferol and quercetin

**Fig. 5:**
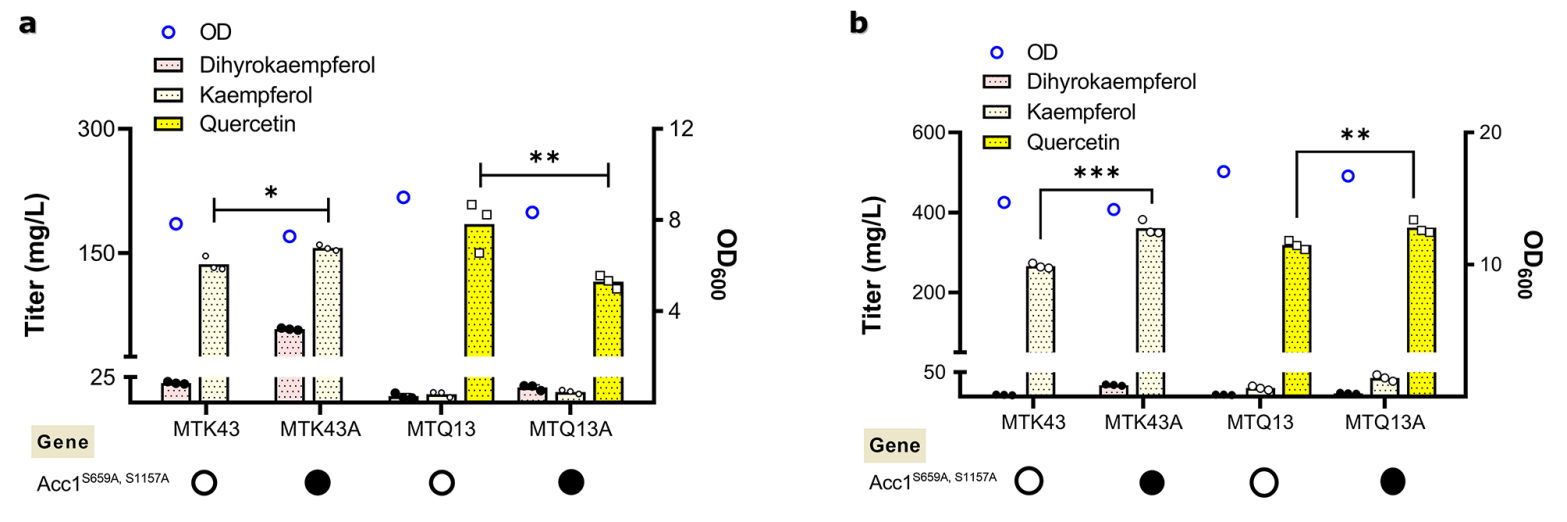
Effect of improved malonyl-CoA supply on production of kaempferol and quercetin. (**a**) Production of kaempferol, quercetin and dihydrokaempferol in different yeast strains. Cells were cultured in a defined minimal medium in shake flask conditions, and samples were taken after 72 h of growth for quercetin, dihydrokaempferol, and kaempferol detection. (**b**) Related yeast strains have been tested in defined minimal medium in shake flask with six discs of FeedBeads, releasing glucose slowly and constantly, instead of full glucose supplement. Samples were taken after 96 h of growth for naringenin, dihydrokaempferol, and kaempferol measurements. The black circle shows the existence of the gene in the strain, instead of the white circle representing its absence. Statistical analysis was performed by using one-way ANOVA, Tukey’s multiple comparison test (* < 0.05, ** < 0.01, *** < 0.001). All data represent the mean ± standard deviation of 3 biologically independent samples

Both kaempferol and quercetin production were significantly increased under fed batch like conditions which prevents overflow metabolism by slow release of glucose. This beneficial effect of fed-batch-like condition has been shown before to further increase naringenin production with enhanced malonyl-CoA supply in NAG3-4 [31], which led us to consider another precursor malonyl-CoA for naringenin biosynthesis. Thus, we overexpressed a mutant version of acetyl-CoA carboxylase (Acc1^S659A, S1157A^), which demonstrated improved activity [41] and increased malonyl-CoA supply [43], in our best kaempferol producer MTK43 and quercetin producer MTQ13. Under normal batch conditions, while production of kaempferol slightly increased quercetin production was significantly reduced (Fig. 5a). This was not surprisingly as it was seen in previous study [31], in which under normal batch condition improving malonyl-CoA supply in NAG3-4 did not significantly increase the production of naringenin, key precursor for kaempferol and quercetin. The reason might be attributed to the imbalance of malonyl-CoA and *p*-coumaric acid supply, especially for quercetin production the biomass was significantly decreased when improving malonyl-CoA supply under batch condition (Fig. 5a). However, under fed batch like conditions overexpressing Acc1 mutant resulted in higher titers for both kaempferol (347 mg L^-1^ in MTK43A) and quercetin (355 mg L^-1^ in MTQ13A), representing 36% and 12% improvement compared to the counter strains, respectively (Fig. 5b). This agreed well with previous study [31] that fed batch mimicking condition was beneficial to naringenin production and therefore downstream products such as kaempferol and quercetin.

**Fig. 6:**
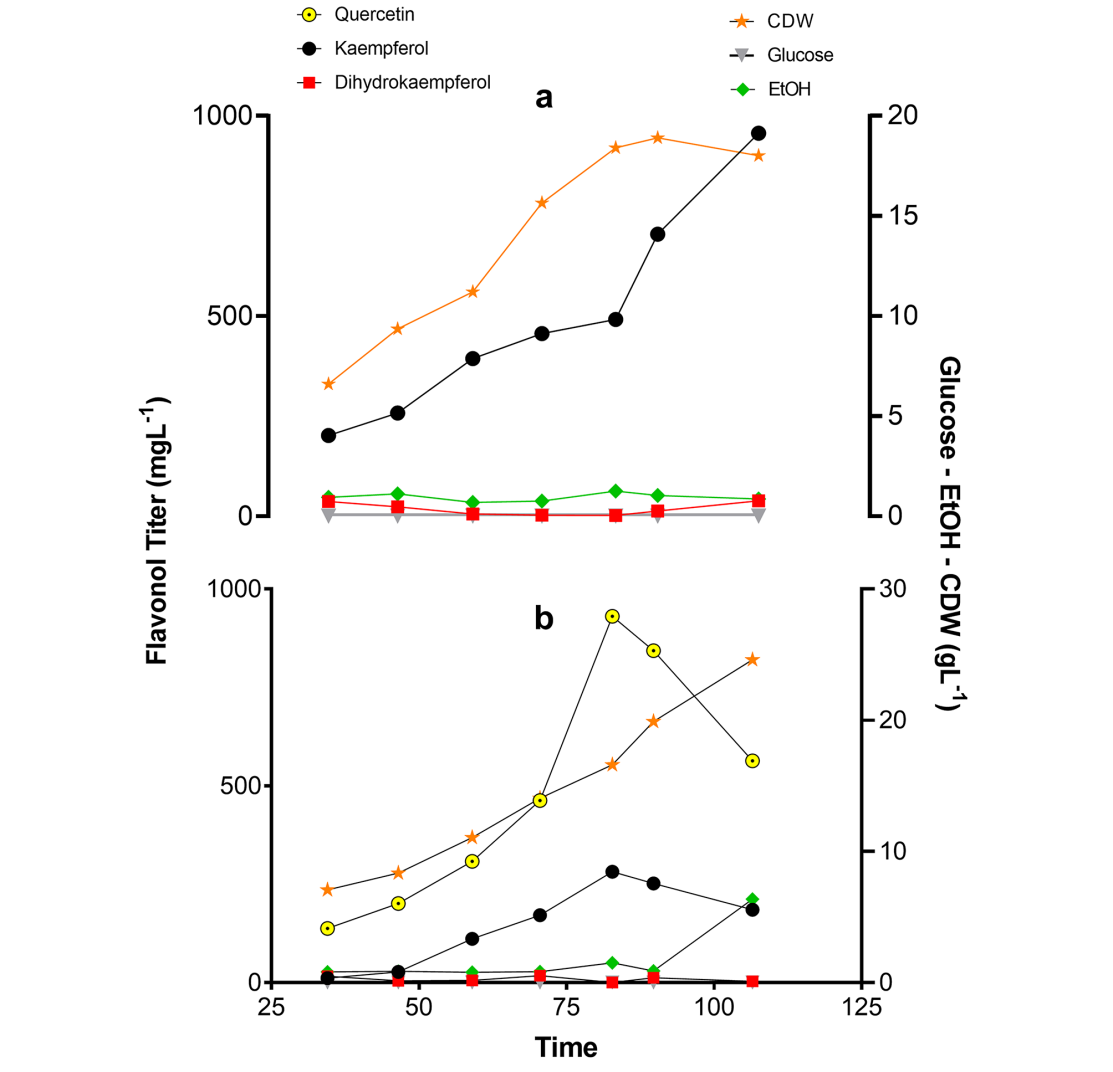
Fed batch fermentation of strain MTK43A (**a**) and MTQ13A (**b**) for kaempferol and quercetin production. Quercetin, kaempferol and dihydrokaempferol, ethanol (EtOH), glucose and cell dry weight (CDW) are shown during glucose-limited controlled fed batch cultivations. The cultivations were performed at least in triplicates, here a representative graph is shown for each strain

To further evaluate the performance of our engineered strains, glucose-limited fed-batch fermentation was conducted. The final kaempferol producer MTK43A has reached a titer of 956 mg L^− 1^, with a very low amount of dihydrokaempferol (about 40 mg L^− 1^) in the whole fed batch cultivations (Fig. 6a). On the other hand, the final quercetin strain MTQ13A achieved 930 mg L^− 1^ of quercetin production at the best (Fig. 6b), but kaempferol accumulation was observed in the same trend of quercetin formation, which indicates the limitation of downstream of kaempferol in the final strain. Current fed batch processes were not optimal, as can be seen (Fig. 6) that ethanol started to be accumulated, which indicates the process is not under glucose-limiting condition (pure respiratory metabolism). Furthermore, the final biomass achieved was much lower in kaempferol than that in quercetin production. Although the fermentation conditions were not optimized, both kaempferol and quercetin production achieved in this study are highest titers reported to date in microbial cell factories (Table 1). Future studies on fermentation process development, i.e., media compositions, oxygen supply and feeding strategies, are required to improve yields and productivities for commercial production.

**Table 1:** Comparison of engineered yeast for production of kaempferol and quercetin

Condition	Target compound	Titer (mg L^− 1^)		Cultivation Time (h)	Origin
Shake flask	Kaempferol	4,6		70	[25]
		26,6		72	[26]
		86		72	[27]
		156		72	This study
FeedBeads^*^	Kaempferol	360		96	This study
Fed-batch	Kaempferol	66,3		48	[28)
		956		106	This study
Shake flask	Quercetin	0,4		70	[25]
		20,4		72	[26]
		194		72	This study
FeedBeads^*^	Quercetin	362		96	This study
Fed-batch	Quercetin	930		82	This study

* Slow release of glucose from elastomer tablets in shake flask conditions.

## Conclusion

In this study, *S. cerevisiae* was engineered for high production of kaempferol and quercetin in minimal media from glucose. First, the kaempferol biosynthetic pathway was reconstructed via screening various F3H and FLS enzymes, from which AtF3H and AtFLS were determined as the best combination. In addition, we demonstrated that amplification of the rate limiting enzyme AtFLS could reduce the dihydrokaempferol accumulation and improve kaempferol production. Increasing the availability of precursor malonyl-CoA further improved the production of kaempferol and quercetin under fed-batch mimicking conditions. Furthermore, the use of slow release of glucose, for instance, FeedBeads, significantly improved both kaempferol and quercetin production. In summary, a high amount of 956 mg L^− 1^ of kaempferol and 930 mg L^− 1^ of quercetin was achieved in yeast via *de novo* biosynthesis, which are among the highest production titers reported for kaempferol and quercetin. Our work provides a promising platform for sustainable and scalable production of kaempferol, quercetin and compounds derived thereof.

## Electronic supplementary material

Below is the link to the electronic supplementary material.

**Additional file 1:** **Table S1**. Plasmids used in this study. **Table S2**. S.cerevisiae strains used in this study. **Table S3.** Codon optimized genes used in this study. **Table S4**. Oligonucleotides used in this study. **Table S5.** Constructed DNA modules in this study. **Table S6**. Homology sequences for integration sites at S. cerevisiae chromosome.

**Additional file 2:** Feeding profile for glucose limited fed-batch fermentation of kaempferol and quercetin.

## Data Availability

The datasets used and analyzed during the current study are available from the corresponding author on reasonable request.
