# Supplementary material for: Optimizing yeast for high-level production of kaempferol and quercetin
Source: Microb Cell Fact. 2023 Apr 20;22:74. doi: 10.1186/s12934-023-02084-4 (PMC10116799; doi:10.1186/s12934-023-02084-4)
Supplement: Supplementary file 1 — Additional file 1: Table S1. Plasmids used in this study. Table S2. S.cerevisiae strains used in this study. Table S3. Codon optimized genes used in this study. Table S4. Oligonucleotides used in this study. Table S5. Constructed DNA modules in this study. Table S6. Homology sequences for integration sites at S. cerevisiae chromosome. [file 12934_2023_2084_MOESM1_ESM.docx]

# SUPPLEMENTARY DATA

**Table 1.** Plasmids used in this study.

| **Plasmid ID** | **Characteristic** | **Origin** |
| --- | --- | --- |
| pMT1 | Template for OsF3H | This study |
| pMT2 | Template for PdFLS | This study |
| pMT3 | Template for VvFLS | This study |
| pSY31 | Template for AtF3H | This study |
| pSY34 | Template for AtFLS | This study |
| pSY38 | Template for GmFMO | This study |
| pSY39 | Template for AtFMO | This study |
| pSY40 | Template for PhFMO | This study |
| pAD | Template for Acc1 | (Shi et al., 2014) |
| **gRNA vectors** | | |
| pMEL10 | 2μm ampR KlURA3 gRNA-CAN1.Y | (Mans et al. 2015) |
| pQC006 | 2μm ampR KlURA3 gRNA-XI-3.Y | (Liu et al. 2019) |
| pQC009 | 2μm ampR KlURA3 gRNA-XI-5.Y | (Liu et al. 2019) |
| pQC029 | 2μm ampR URA3 gRNA-X-2.Y [2x] | (Liu et al. 2019) |
| pQC130 | 2μm ampR URA3 gRNA-XI-2.Y [2x] | (Liu et al. 2019) |

**Table 2.** *S. cerevisiae* strains used in this study

| **Strain ID** | **Relevant Genotype** | **Parental Strain** | **Origin** |
| --- | --- | --- | --- |
| IMX581 | MATa ura3-52 can1∆::cas9-natNT2 TRP1 LEU2 HIS3 |  | (Mans et al. 2015) |
| NAG10 | MATa ura3-52 can1∆::cas9-natNT2 TRP1 LEU2 HIS3 ARO4*, ARO7*, **X 3::**EcaroL, ARO1, ARO2, ARO3, **XII-2::** PAL2, C4H, ATR2, CYB5, **XII-4::** 4CL, CHS, CHI | IMX581 | (Mao et al. 2022) |
| NAG 3-4 | MATa ura3-52 can1∆::cas9-natNT2 TRP1 LEU2 HIS3 ARO4*, ARO7*, **X 3::**EcaroL, ARO1, ARO2, ARO3, **XII-2::** PAL2, C4H, ATR2, CYB5, **XII-4::** 4CL, CHS, CHI, **XII-1::** 4CL, CHS, CHI, **XII-5::** 4CL, CHS, CHI, **XI-1::** CHS, CHI | NAG10 | (Mao et al. 2022) |
| KB1 | MATa ura3-52 can1∆::cas9-natNT2 TRP1 LEU2 HIS3 ARO4*, ARO7*, **X 3::**EcaroL, ARO1, ARO2, ARO3, **XII-2::** PAL2, C4H, ATR2, CYB5, **XII-4::** 4CL, CHS, CHI, **XI-3::**OsF3H, AtFLS | NAG10 | This study |
| KB2 | MATa ura3-52 can1∆::cas9-natNT2 TRP1 LEU2 HIS3 ARO4*, ARO7*, **X 3::**EcaroL, ARO1, ARO2, ARO3, **XII-2::** PAL2, C4H, ATR2, CYB5, **XII-4::** 4CL, CHS, CHI, **XI-3::**OsF3H, PdFLS | NAG10 | This study |
| KB3 | MATa ura3-52 can1∆::cas9-natNT2 TRP1 LEU2 HIS3 ARO4*, ARO7*, **X 3::**EcaroL, ARO1, ARO2, ARO3, **XII-2::** PAL2, C4H, ATR2, CYB5, **XII-4::** 4CL, CHS, CHI, **XI-3::**OsF3H, VvFLS | NAG10 | This study |
| KB4 | MATa ura3-52 can1∆::cas9-natNT2 TRP1 LEU2 HIS3 ARO4*, ARO7*, **X 3::**EcaroL, ARO1, ARO2, ARO3, **XII-2::** PAL2, C4H, ATR2, CYB5, **XII-4::** 4CL, CHS, CHI, **XI-3::**AtF3H, AtFLS | NAG10 | This study |
| KB5 | MATa ura3-52 can1∆::cas9-natNT2 TRP1 LEU2 HIS3 ARO4*, ARO7*, **X 3::**EcaroL, ARO1, ARO2, ARO3, **XII-2::** PAL2, C4H, ATR2, CYB5, **XII-4::** 4CL, CHS, CHI, **XI-3::**AtF3H, PdFLS | NAG10 | This study |
| KB6 | MATa ura3-52 can1∆::cas9-natNT2 TRP1 LEU2 HIS3 ARO4*, ARO7*, **X 3::**EcaroL, ARO1, ARO2, ARO3, **XII-2::** PAL2, C4H, ATR2, CYB5, **XII-4::** 4CL, CHS, CHI, **XI-3::**AtF3H, VvFLS | NAG10 | This study |
| MTK1 | MATa ura3-52 can1∆::cas9-natNT2 TRP1 LEU2 HIS3 ARO4*, ARO7*, **X 3::**EcaroL, ARO1, ARO2, ARO3, **XII-2::** PAL2, C4H, ATR2, CYB5, **XII-4::** 4CL, CHS, CHI, **XII-1::** 4CL, CHS, CHI, **XII-5::** 4CL, CHS, CHI, **XI-1::** CHS, CHI, **XI-3::**OsF3H, AtFLS | NAG 3-4 | This study |
| MTK2 | MATa ura3-52 can1∆::cas9-natNT2 TRP1 LEU2 HIS3 ARO4*, ARO7*, **X 3::**EcaroL, ARO1, ARO2, ARO3, **XII-2::** PAL2, C4H, ATR2, CYB5, **XII-4::** 4CL, CHS, CHI, **XII-1::** 4CL, CHS, CHI, **XII-5::** 4CL, CHS, CHI, **XI-1::** CHS, CHI, **XI-3::**OsF3H, PdFLS | NAG 3-4 | This study |
| MTK3 | MATa ura3-52 can1∆::cas9-natNT2 TRP1 LEU2 HIS3 ARO4*, ARO7*, **X 3::**EcaroL, ARO1, ARO2, ARO3, **XII-2::** PAL2, C4H, ATR2, CYB5, **XII-4::** 4CL, CHS, CHI, **XII-1::** 4CL, CHS, CHI, **XII-5::** 4CL, CHS, CHI, **XI-1::** CHS, CHI, **XI-3::**OsF3H, VvFLS | NAG 3-4 | This study |
| MTK4 | MATa ura3-52 can1∆::cas9-natNT2 TRP1 LEU2 HIS3 ARO4*, ARO7*, **X 3::**EcaroL, ARO1, ARO2, ARO3, **XII-2::** AtPAL2, AtC4H, ATR2, CYB5, **XII-4::** At4CL, CHS, CHI, **XII-1::** 4CL, CHS, CHI, **XII-5::** 4CL, CHS, CHI, **XI-1::** CHS, CHI, **XI-3::**AtF3H, AtFLS | NAG 3-4 | This study |
| MTK5 | MATa ura3-52 can1∆::cas9-natNT2 TRP1 LEU2 HIS3 ARO4*, ARO7*, **X 3::**EcaroL, ARO1, ARO2, ARO3, **XII-2::** PAL2, C4H, ATR2, CYB5, **XII-4::** 4CL, CHS, CHI, **XII-1::** 4CL, CHS, CHI, **XII-5::** 4CL, CHS, CHI, **XI-1::** CHS, CHI, **XI-3::**AtF3H, PdFLS | NAG 3-4 | This study |
| MTK6 | MATa ura3-52 can1∆::cas9-natNT2 TRP1 LEU2 HIS3 ARO4*, ARO7*, **X 3::**EcaroL, ARO1, ARO2, ARO3, **XII-2::** PAL2, C4H, ATR2, CYB5, **XII-4::** 4CL, CHS, CHI, **XII-1::** 4CL, CHS, CHI, **XII-5::** 4CL, CHS, CHI, **XI-1::** CHS, CHI, **XI-3::**AtF3H, VvFLS | NAG 3-4 | This study |
| MTK42 | MATa ura3-52 can1∆::cas9-natNT2 TRP1 LEU2 HIS3 ARO4*, ARO7*, **X 3::**EcaroL, ARO1, ARO2, ARO3, **XII-2::** AtPAL2, AtC4H, ATR2, CYB5, **XII-4::** At4CL, CHS, CHI, **XII-1::** 4CL, CHS, CHI, **XII-5::** 4CL, CHS, CHI, **XI-1::** CHS, CHI, **XI-3::**AtF3H, AtFLS, **X-2::**AtFLS | MTK4 | This study |
| MTK43 | MATa ura3-52 can1∆::cas9-natNT2 TRP1 LEU2 HIS3 ARO4*, ARO7*, **X 3::**EcaroL, ARO1, ARO2, ARO3, **XII-2::** AtPAL2, AtC4H, ATR2, CYB5, **XII-4::** At4CL, CHS, CHI, **XII-1::** 4CL, CHS, CHI, **XII-5::** 4CL, CHS, CHI, **XI-1::** CHS, CHI, **XI-3::**AtF3H, AtFLS, **X-2::**FLS**, XI-2::**AtFLS | MTK42 | This study |
| MTQ1 | MATa ura3-52 can1∆::cas9-natNT2 TRP1 LEU2 HIS3 ARO4*, ARO7*, **X 3::**EcaroL, ARO1, ARO2, ARO3, **XII-2::** AtPAL2, AtC4H, ATR2, CYB5, **XII-4::** At4CL, CHS, CHI, **XII-1::** 4CL, CHS, CHI, **XII-5::** 4CL, CHS, CHI, **XI-1::** CHS, CHI, **XI-3::**AtF3H, AtFLS, **XI-5::**GmFMO | MTK4 | This study |
| MTQ2 | MATa ura3-52 can1∆::cas9-natNT2 TRP1 LEU2 HIS3 ARO4*, ARO7*, **X 3::**EcaroL, ARO1, ARO2, ARO3, **XII-2::** AtPAL2, AtC4H, ATR2, CYB5, **XII-4::** At4CL, CHS, CHI, **XII-1::** 4CL, CHS, CHI, **XII-5::** 4CL, CHS, CHI, **XI-1::** CHS, CHI, **XI-3::**AtF3H, AtFLS, **XI-5::**AtFMO | MTK4 | This study |
| MTQ3 | MATa ura3-52 can1∆::cas9-natNT2 TRP1 LEU2 HIS3 ARO4*, ARO7*, **X 3::**EcaroL, ARO1, ARO2, ARO3, **XII-2::** AtPAL2, AtC4H, ATR2, CYB5, **XII-4::** At4CL, CHS, CHI, **XII-1::** 4CL, CHS, CHI, **XII-5::** 4CL, CHS, CHI, **XI-1::** CHS, CHI, **XI-3::**AtF3H, AtFLS, **XI-5::**PhFMO | MTK4 | This study |
| MTQ13 | MATa ura3-52 can1∆::cas9-natNT2 TRP1 LEU2 HIS3 ARO4*, ARO7*, **X 3::**EcaroL, ARO1, ARO2, ARO3, **XII-2::** AtPAL2, AtC4H, ATR2, CYB5, **XII-4::** At4CL, CHS, CHI, **XII-1::** 4CL, CHS, CHI, **XII-5::** 4CL, CHS, CHI, **XI-1::** CHS, CHI, **XI-3::**AtF3H, AtFLS, **X-2::**FLS**, XI-2::**FLS, **XI-5::**GmFMO | MTK42 | This study |
| MTQ23 | MATa ura3-52 can1∆::cas9-natNT2 TRP1 LEU2 HIS3 ARO4*, ARO7*, **X 3::**EcaroL, ARO1, ARO2, ARO3, **XII-2::** AtPAL2, AtC4H, ATR2, CYB5, **XII-4::** At4CL, CHS, CHI, **XII-1::** 4CL, CHS, CHI, **XII-5::** 4CL, CHS, CHI, **XI-1::** CHS, CHI, **XI-3::**AtF3H, AtFLS, **X-2::**FLS**, XI-2::**FLS, **XI-5::**AtFMO | MTK42 | This study |
| MTK43A | MATa ura3-52 can1∆::cas9-natNT2 TRP1 LEU2 HIS3 ARO4*, ARO7*, **X 3::**EcaroL, ARO1, ARO2, ARO3, **XII-2::** AtPAL2, AtC4H, ATR2, CYB5, **XII-4::** At4CL, CHS, CHI, **XII-1::** 4CL, CHS, CHI, **XII-5::** 4CL, CHS, CHI, **XI-1::** CHS, CHI, **XI-3::**AtF3H, AtFLS, **X-2::**FLS**, XI-2::**AtFLS **V**:: ACC1^S659A,S1157A^ | MTK43 | This study |
| MTQ13A | MATa ura3-52 can1∆::cas9-natNT2 TRP1 LEU2 HIS3 ARO4*, ARO7*, **X 3::**EcaroL, ARO1, ARO2, ARO3, **XII-2::** AtPAL2, AtC4H, ATR2, CYB5, **XII-4::** At4CL, CHS, CHI, **XII-1::** 4CL, CHS, CHI, **XII-5::** 4CL, CHS, CHI, **XI-1::** CHS, CHI, **XI-3::**AtF3H, AtFLS, **X-2::**FLS**, XI-2::**FLS, **XI-5::**GmFMO **V**:: ACC1^S659A,S1157A^ | MTQ13 | This study |

**Table 3.** Codon optimized genes used in this study.

| **Gene** | **Name** | **Source** | **Sequence (5’-3’)** |
| --- | --- | --- | --- |
| OsF3H | Flavanone 3-hydroxylase | *Oryza sativa* | ATGGCACCAGTTGCTACTACATTTTTGCCAACTGCTTCTAATGAAGCAACATTAAGACCATCATTTGTTAGAGATGAAGATGAAAGACCAAGAGTTGCATACAACCAATTTTCTGATGCTGTTCCAGTTATTTCATTGCAAGGTATTGATGAAGCTGCAAGAGCTGAAATTAGAGCAAGAGTTGCTGGTGCATGTGAAGAATGGGGTATTTTTCAAGTTGTTGATCATGGTGTTGATGCAGGTTTGGTTGCAGATATGGCTAGATTGGCTAGAGATTTCTTTGCTTTGCCACCAGAAGATAAATTGAGATTTGATATGTCTGGTGGTAAAAAGGGTGGTTTTATTGTTTCTTCACATTTGCAAGGTGAAGCTGTTAAGGATTGGAGAGAAATCGTTACTTACTTCTCATACCCTGTTAAGTCAAGAGATTATTCAAGATGGCCAGATAAACCAGCTGGTTGGAGAGCAGTTGTTGAACAATACTCTGAAAGATTGATGGGTTTAGCATGTAAATTGTTGGGTGTTTTGTCAGAAGCTATGGGTTTGGATACAAATGCTTTAGCAGATGCTTGTGTTGATATGGATCAAAAGGTTGTTGTTAACTTCTACCCAAAGTGTCCACAACCAGATTTGACTTTGGGTTTAAAAAGACATACAGATCCAGGTACTATCACATTGTTGTTGCAAGATTTGGTTGGTGGTTTACAAGCTACTAGAGATGCAGGTAAAACTTGGATTACAGTTCAACCAATTCCAGGTTCTTTTGTTGTTAATTTGGGTGACCATGCTCATTACTTATCAAACGGTAGATTCAAAAACGCTGATCATCAAGCAGTTGTTAATTCTGATTGTTGTAGATTGTCAATCGCAACTTTCCAAAATCCAGCTCCAGATGCAATGGTTTATCCATTGGCTGTTAGAGATGGTGAAGAACCAATTTTAGAAGAACCAATCACATTCGCTGAAATGTACAGAAGAAAAATGGCTAGAGATTTGGAATTAGCAAAATTGAAAAAGAAAGCTAAAGAACAAAGACAATTGCAACAAGCTGCATTGCCACCACCACCACCAACACAAGTTGCTGCAGAATTAGCTGCACAAAAACCAAAATCTTTGGATGAAATTTTAGCT |
| AtF3H |  | *Arabidopsis thaliana* | ATGGCTCCAGGTACTTTGACAGAATTAGCTGGTGAATCTAAGTTGAACTCAAAGTTCGTTAGAGATGAAGATGAAAGACCAAAGGTTGCTTACAACGTTTTCTCTGATGAAATCCCAGTTATCTCATTAGCAGGTATTGATGATGTTGATGGCAAGAGAGGTGAAATCTGTAGACAAATCGTTGAAGCTTGTGAAAATTGGGGTATTTTTCAAGTTGTTGATCATGGTGTTGATACTAATTTGGTTGCAGATATGACAAGATTAGCTAGAGATTTCTTTGCATTGCCACCAGAGGATAAGTTGAGATTCGATATGTCTGGTGGTAAAAAAGGTGGTTTCATCGTTTCTTCACATTTGCAAGGTGAAGCTGTTCAAGATTGGAGAGAAATCGTTACTTACTTCTCTTACCCAGTTAGAAACAGAGATTATTCAAGATGGCCAGATAAACCAGAAGGTTGGGTTAAGGTTACAGAAGAATACTCTGAAAGATTGATGTCATTGGCTTGTAAGTTGTTGGAAGTTTTGTCTGAAGCAATGGGTTTGGAAAAGGAATCATTGACTAACGCATGTGTTGATATGGATCAAAAGATCGTTGTTAACTACTACCCAAAATGTCCACAACCAGATTTGACTTTGGGTTTAAAGAGACATACAGATCCAGGTACTATCACATTGTTGTTGCAAGATCAAGTTGGTGGTTTGCAAGCTACAAGAGATAATGGTAAAACTTGGATTACAGTTCAACCAGTTGAAGGTGCATTTGTTGTTAATTTGGGTGATCATGGTCATTTCTTGTCTAACGGTAGATTCAAGAACGCTGATCATCAAGCAGTTGTTAACTCTAACTCTTCAAGATTGTCAATTGCTACTTTTCAAAATCCAGCTCCAGATGCAACAGTTTATCCATTGAAAGTTAGAGAAGGTGAAAAGGCTATCTTGGAAGAACCAATCACTTTCGCAGAAATGTACAAGAGAAAGATGGGTAGAGATTTGGAATTGGCTAGATTGAAAAAATTAGCAAAGGAAGAAAGAGATCATAAGGAAGTTGATAAGCCAGTTGATCAAATTTTTGCTTAA |
| AtFLS | Flavonol synthase | *Arabidopsis thaliana* | ATGGAAGTTGAAAGAGTTCAAGATATCTCTTCATCTTCATTGTTGACTGAAGCTATCCCATTGGAATTCATCAGATCTGAAAAGGAACAACCAGCTATCACTACATTCAGAGGTCCAACACCAGCAATTCCAGTTGTTGATTTGTCTGATCCAGATGAAGAATCAGTTAGAAGAGCTGTTGTTAAAGCATCAGAAGAATGGGGTTTATTCCAAGTTGTTAACCATGGTATCCCAACTGAATTGATCAGAAGATTGCAAGATGTTGGTAGAAAGTTTTTCGAATTGCCATCTTCAGAAAAAGAATCTGTTGCTAAACCAGAAGATTCAAAGGATATCGAAGGTTACGGTACAAAGTTGCAAAAGGATCCAGAAGGTAAAAAAGCATGGGTTGATCATTTGTTCCATAGAATCTGGCCACCATCTTGTGTTAACTACAGATTCTGGCCAAAGAACCCACCAGAATACAGAGAAGTTAACGAAGAATACGCAGTTCATGTTAAAAAATTGTCTGAAACTTTGTTGGGTATCTTGTCAGATGGTTTGGGTTTAAAGAGAGATGCTTTGAAGGAAGGTTTAGGTGGTGAAATGGCAGAATACATGATGAAGATCAACTATTACCCACCATGTCCAAGACCAGATTTGGCTTTAGGTGTTCCAGCACATACTGATTTGTCTGGTATTACATTGTTAGTTCCAAACGAAGTTCCAGGTTTACAAGTTTTCAAGGATGATCATTGGTTTGATGCTGAATACATCCCATCTGCAGTTATCGTTCATATCGGTGATCAAATCTTGAGATTGTCAAACGGTAGATACAAGAACGTTTTGCATAGAACTACAGTTGATAAGGAAAAGACTAGAATGTCTTGGCCAGTTTTCTTGGAACCACCAAGAGAAAAAATTGTTGGTCCATTGCCAGAATTAACAGGTGATGATAACCCACCAAAGTTCAAGCCATTTGCTTTTAAAGATTATTCATACAGAAAATTGAATAAATTGCCATTAGATTAA |
| PdFLS |  | *Populus deltoides* | ATGGAATTTGATAGAGTTCAAGCTATCGCATCTTTATCATTTGATAAGGAAACTATCCCAGAAGAGTTTATTAGACCAGAAAAAGAACAACCAGCTGCAACTACATTTCATGGTCCAGTTCCAGAAATCCCAACAATCGATTTGAACGATCCAAACCCAGAAAATTTGGTTAGATTGATCGCTGATGCATCTAAGGAATGGGGTATCTTCCAAGTTGTTAACCATGGTATTCCATCAGATTTGATTGCTAAATTGCAAGATGTTGGTAAAAAGTTTTTCGAATTGCCACAAGAAGAAAAGGAAGTTTACGCAAAGCCACATGATTCTAAGTCAATCGAAGGTTACGGTTCTAAATTGCAAAACAACCCACAAGTTAAGAAATCTTGGGTTGATCATTTGTTCCATATTATTTGGCCACCATCTTCAATTAATTACCAATTCTGGCCAAACAATCCACCATCTTACAGAGAAGTTAACGAAGAATACGCTAAGTACATGAGAGAAGTTACTGATAAATTGTTTACAGCATTGTCATTAGGTTTGGGTTTAGAAGGTCATGCTTTAAAAGAAGGTGCAGGTGGTGAAGAAATCGAATACATGTTGAAGATTAATTACTACCCACCATGTCCAAGACCAGATTTGACTTTAGGTGTTGCTGCACATACTGATTTGTCTGCTTTGACAATCTTGGTTCCAAACGAAGTTCCAGGTTTGCAAATTTTTAAGGATGGTAACTGGTTCGAGGCTAAGTACATCCCAAACGCATTGATCATCCATATCGGTGACCAAATCGAAATCTTGTCTAACGGTAAATACAAAGCTGTTTTGCATAGAACTACAGTTGCTAAAGATAAAGCAAGAATGTCATGGCCAGTTTTCTTGGAACCACCAGGCGAATTGGTTGTTGGTCCATTGCCACATTTGATTAATGAAGATAATCCACCAAAGTTTAAAGCTAAGAAATTCGAAGATTATATGTACTGTAAATTGAATAGATTGCCACAA |
| VvFLS |  | *Vitis Vinifera* | ATGGATATGGAAAGAGTTCAAGCTATCGCATTTTCTTTTATGACTGAAGGTAGAATACCACCAGAGTTTATTAGATCAGAAAAAGAACAACCAGCTATTACTACATTGCATGGTTACGATCCACAAATCCCAACTATCGATTTTTCTGATCCAGATGAAGATTCATTGACAAGATTAATTGCTGAAGCTAGTAGAGATTGGGGCATGTTCCAAATCGTTAACCATGGTATTCCAACTCATGTTATTAATAATTTGCAAAAGGTTGGTGAAGATTTCTTTGCTTTGCCACAAGTTGAAAAGGAATTGTACGCAAAGCCACCAGATTCTAAGTCAATTGAAGGTTACGGTACAAGATTGCAAAAAGAAGAAGAAGGTAAAAGAGCTTGGGTTGATCATTTGTTCCATAAGATCTGGCCACCATCTGCAATTAATTACCAATTCTGGCCTAAAAATCCACCATCATATAGAGATGCTAATGAAGTTTACGCAAAATGTTTGAGAGGTGTTGCTGATAAATTGTTTTCAAGATTGTCAGTTGGTTTGGGTTTAGGTGAAAAAGAATTGAGAGAATCTGTTGGTGGTGACGAATTGACATACTTGTTGAAGATTAATTACTACCCACCATGTCCAAGACCAGATTTGGCTTTAGGTGTTGTTGCACATACTGATATGTCAGCTATCACAATCTTAGTTCCAAACCATGTTCAAGGTTTGCAATTGTTTAGAGACGATCATTGGTTCGATGTTAAGTACATTCCAAATGCATTGGTTATTCATATCGGTGACCAATTGGAAATCTTGTCTAACGGTAAATACAAATCAGTTTTGCATAGAACTACAGTTAAGAAAGATATGACTAGAATGTCTTGGCCAGTTTTCTTGGAACCACCACCAGAATTAGCAATTGGTCCATTGCCAAAATTGACATCAGAAGATAATCCACCAAAATATAAAAAGAAAAGATACTGTGATTATGTTTACTGTAAATTGAATAAGATTCCACAA |
| GmFMO | flavin-containing monooxygenase | *Glycine max* | ATGTCTCCATTAATTGTTGCTTTGGCAACTATTGCTGCAGCTATCTTGATCTACAGAATCATCAAGTTCATTACAAGACCATCATTACCATTGCCACCAGGTCCAAAACCATGGCCAATTGTTGGTAATTTACCACATATGGGTCCAGTTCCACATCATTCTTTGGCAGCTTTAGCTAGAATTCATGGTCCATTGATGCATTTAAGATTGGGTTTTGTTGATGTTGTTGTTGCAGCTTCTGCATCAGTTGCTGAACAATTCTTGAAGATCCATGATTCTAACTTCTCTTCAAGACCACCAAACGCTGGTGCAAAGTACATCGCATACAACTACCAAGATTTGGTTTTTGCTCCATACGGTCCAAGATGGAGATTGTTGAGAAAGTTGACTTCTGTTCATTTGTTTTCAGGCAAGGCAATGAACGAATTCAGACATTTGAGACAAGAAGAAGTTGCTAGATTGACTTGTAACTTGGCATCTTCAGATACAAAGGCTGTTAACTTGGGTCAATTGTTGAACGTTTGTACTACAAATGCTTTGGCAAGAGCTATGATCGGTAGAAGAGTTTTCAACGATGGTAACGGTGGTTGTGATCCAAGAGCAGATGAATTCAAGGCTATGGTTATGGAAGTTATGGTTTTGGCTGGTGTTTTCAACATCGGTGATTTCATCCCATCATTGGAATGGTTGGATTTGCAAGGTGTTCAAGCAAAGATGAAGAAGTTGCATAAGAGATTCGATGCATTTTTGACATCTATTATTGAAGAACATAATAATTCTTCATCTAAAAATGAAAATCATAAAAATTTCTTGTCTATTTTGTTGTCATTGAAGGATGTTAGAGATGATCATGGTAACCATTTGACTGATACAGAAATCAAGGCATTGTTGTTGAACATGTTCACTGCTGGTACTGATACATCATCTTCAACTACAGAATGGGCAATCGCTGAATTGATCAAGAACCCACAAATCTTGGCTAAGTTGCAACAAGAATTGGATACAGTTGTTGGTAGAGATAGATCAGTTAAGGAAGAAGATTTGGCACATTTGCCATACTTGCAAGCTGTTATCAAGGAAACTTTCAGATTGCATCCATCTACACCATTGTCAGTTCCAAGAGCAGCTGCAGAATCTTGTGAAATCTTCGGTTACCATATCCCAAAGGGTGCAACTTTGTTGGTTAACATCTGGGCAATTGCTAGAGATCCAAAGGAATGGAACGATCCATTGGAATTCAGACCAGAAAGATTTTTATTGGGTGGTGAAAAAGCTGATGTTGATGTTCGTGGTAACGATTTCGAAGTTATCCCATTTGGTGCAGGTAGAAGAATTTGTGCTGGTTTATCTTTGGGTTTACAAATGGTTCAATTATTGACTGCTGCATTAGCTCATTCATTTGATTGGGAATTGGAAGATTGTATGAACCCAGAAAAGTTGAACATGGATGAAGCATACGGTTTGACATTACAAAGAGCTGTTCCATTATCTGTTCATCCAAGACCAAGATTGGCTCCACATGTTTACTCAATGTCTTCATAA |
| AtFMO |  | *Arabidopsis thaliana* | ATGGCTACTTTATTTTTGACAATCTTGTTGGCAACTGTTTTATTTTTGATCTTGAGAATTTTCTCTCATAGAAGAAACAGATCACATAACAACAGATTACCACCAGGTCCAAATCCATGGCCAATTATTGGTAATTTACCACATATGGGTACTAAACCACATAGAACATTGTCTGCTATGGTTACTACATACGGTCCAATCTTGCATTTGAGATTGGGTTTCGTTGATGTTGTTGTTGCTGCATCTAAATCAGTTGCAGAACAATTCTTGAAGATCCATGATGCTAACTTTGCATCTAGACCACCAAATTCAGGTGCTAAGCATATGGCATACAACTACCAAGATTTGGTTTTTGCTCCATACGGTCATAGATGGAGATTGTTGAGAAAGATCTCTTCAGTTCATTTATTTTCAGCTAAGGCATTGGAAGATTTCAAGCATGTTAGACAAGAAGAAGTTGGTACTTTAACAAGAGAATTGGTTAGAGTTGGTACTAAGCCAGTTAACTTGGGTCAATTGGTTAACATGTGTGTTGTTAACGCTTTGGGTAGAGAAATGATTGGTAGAAGATTGTTTGGTGCTGATGCAGATCATAAGGCAGATGAATTCAGATCTATGGTTACAGAAATGATGGCTTTGGCAGGTGTTTTCAACATCGGTGATTTCGTTCCATCATTGGATTGGTTAGATTTGCAAGGTGTTGCTGGCAAGATGAAGAGATTGCATAAGAGATTCGATGCATTTTTATCTTCAATCTTGAAGGAACATGAAATGAACGGTCAAGATCAAAAGCATACTGATATGTTGTCTACATTGATCTCATTGAAGGGTACAGATTTAGATGGTGATGGTGGTTCTTTGACTGATACAGAAATCAAGGCTTTGTTGTTGAACATGTTCACTGCTGGTACTGATACATCTGCATCAACAGTTGATTGGGCTATCGCAGAATTGATCAGACATCCAGATATCATGGTTAAGGCTCAAGAAGAATTGGATATTGTTGTTGGTAGAGATAGACCAGTTAACGAATCTGATATCGCTCAATTGCCATACTTGCAAGCAGTTATCAAGGAAAACTTTAGATTGCATCCACCAACTCCATTATCATTGCCACATATCGCTTCTGAATCATGTGAAATCAACGGTTACCATATCCCAAAGGGTTCTACTTTGTTGACAAACATCTGGGCTATTGCAAGAGATCCAGATCAATGGTCAGATCCATTGGCTTTCAAGCCAGAAAGATTTTTGCCAGGTGGTGAAAAATCTGGTGTTGATGTTAAGGGTTCAGATTTCGAATTGATTCCATTTGGTGCTGGTAGAAGAATTTGTGCAGGTTTATCTTTGGGTTTAAGAACAATCCAATTCTTGACTGCTACATTGGTTCAAGGTTTTGATTGGGAATTAGCAGGTGGTGTTACTCCAGAAAAGTTGAACATGGAAGAATCTTACGGTTTGACATTGCAAAGAGCTGTTCCATTGGTTGTTCATCCAAAACCAAGATTAGCACCAAATGTTTACGGTTTGGGTTCAGGTTAA |
| PhFMO |  | *Petunia hybrida* | ATGGAAATTTTGTCATTAATTTTGTATACTGTTATTTTCTCATTTTTGTTGCAATTCATCTTAAGATCTTTCTTTAGAAAGAGATACCCATTGCCATTGCCACCAGGTCCAAAACCATGGCCAATCATCGGTAACTTGGTTCATTTGGGTCCAAAACCACATCAATCTACTGCTGCAATGGCTCAAACATACGGTCCATTGATGTACTTGAAGATGGGTTTCGTTGATGTTGTTGTTGCTGCATCAGCATCTGTTGCTGCACAATTCTTGAAGACTCATGATGCTAACTTCTCTTCAAGACCACCAAATTCAGGTGCAGAACATATGGCTTACAACTACCAAGATTTGGTTTTCGCACCATATGGTCCAAGATGGAGAATGTTGAGAAAGATCTGTTCAGTTCATTTATTTTCTACAAAGGCTTTGGATGATTTCAGACATGTTAGACAAGATGAAGTTAAGACTTTGACAAGAGCTTTGGCATCTGCTGGTCAAAAACCTGTTAAGTTGGGTCAATTGTTGAACGTTTGTACTACAAACGCATTGGCTAGAGTTATGTTGGGTAAAAGAGTTTTTGCTGATGGTTCTGGTGATGTTGATCCACAAGCTGCAGAATTCAAGTCAATGGTTGTTGAAATGATGGTTGTTGCTGGTGTTTTCAACATCGGTGATTTCATCCCACAATTGAACTGGTTGGATATTCAAGGTGTTGCTGCAAAGATGAAGAAGTTGCATGCAAGATTCGATGCATTTTTGACTGATATCTTGGAAGAACATAAGGGCAAGATCTTTGGTGAAATGAAGGATTTGTTGTCAACATTGATCTCTTTGAAAAATGATGATGCTGATAATGATGGTGGCAAGTTGACTGATACAGAAATCAAGGCATTGTTATTGAATTTGTTTGTTGCTGGTACTGATACATCTTCATCTACAGTTGAATGGGCAATCGCTGAATTGATCAGAAACCCAAAGATTTTGGCACAAGCTCAACAAGAAATCGATAAGGTTGTTGGTAGAGATAGATTGGTTGGTGAATTGGATTTGGCACAATTGACTTACTTGGAAGCTATCGTTAAGGAAACTTTCAGATTGCATCCATCAACACCATTATCTTTGCCAAGAATCGCTTCAGAATCTTGTGAAATCAACGGTTACTTCATCCCAAAGGGTTCTACATTGTTGTTGAACGTTTGGGCAATTGCTAGAGATCCAAATGCATGGGCTGATCCATTGGAATTCAGACCAGAAAGATTTTTGCCAGGTGGTGAAAAACCAAAGGTTGATGTTCGTGGTAACGATTTCGAAGTTATCCCATTTGGTGCAGGTAGAAGAATTTGTGCTGGTATGAACTTGGGTATCAGAATGGTTCAATTGATGATCGCAACTTTGATCCATGCTTTCAACTGGGATTTGGTTTCAGGTCAATTACCAGAAATGTTGAATATGGAAGAAGCATATGGTTTAACATTGCAAAGAGCTGATCCATTAGTTGTTCATCCAAGACCAAGATTGGAAGCACAAGCTTACATTGGTTAA |

**Table 4.** Oligonucleotides were used in this study

| **Name** | **Sequence (5’-3’)** |
| --- | --- |
| XI-3_HR_US_Fw | GGGAGTCA GGTCTATCATCTTG |
| XI-3_HR_US_Rv_with_pTEF1 | GGAAGAGTAAAAAAGGAGTAGAAACATTTTGAAGCTATCGTAGAA ATCAGACGCACG |
| pTEF1_Fw | ATAGCTTCAAAATGTTTCTACTC |
| pTEF1_Rv_with_OsF3H | GTTGGCAAAAATGTAGTAGCAACTGGTGCCATTTTGTAATTAAAACTTAGATTAGATTGC |
| pTEF1_Fw | ATAGCTTCAAAATGTTTCTACTC |
| pTEF1_Rv_with_AtF3H | CCAGCTAATTCTGTCAAAGTACCTGGAGCCATTTTGTAATTAAAACTTAGATTAGATTGC |
| OsF3H_Fw | ATGGCACCAGTTGCTAC |
| OsF3H_Rv | AGCTAAAATTTCATCCAAAGATTTTG |
| AtF3H_Fw | ATGGCTCCAGGTACTTTG |
| AtF3H_Rv | TTAAGCAAAAATTTGATCAACTGG |
| tPRM1_Fw_with_OsF3H | GCTGCACAAAAACCAAAATCTTTGGATGAAATTTTAGCTTAAACAGAAGACGGGAGACAC |
| tPRM1_Rv | ATTTTCAACATCGTATTTTCCGAAG |
| tPRM1_Fw_with_AtF3H | CATAAGGAAGTTGATAAGCCAGTTGATCAAATTTTTGCTTAAACAGAAGACGGGAGACAC |
| tPRM1_Rv | ATTTTCAACATCGTATTTTCCGAAG |
| tTDH2_Fw_with_tPRM1 | CATTATGCAACGCTTCGGAAAATACGATGTTGAAAATGCGAAAAGCCAATTAGTG |
| tTDH2_Rv_with_AtFLS | CATACAGAAAATTGAATAAATTGCCATTAGATTAAATTTAACTCCTTAAGTTACTTTAATG |
| tTDH2_Fw_with_tPRM1 | CATTATGCAACGCTTCGGAAAATACGATGTTGAAAATGCGAAAAGCCAATTAGTG |
| tTDH2_Rv_with_PdFLS | GTACTGTAAATTGAATAGATTGCCACAATAAATTTAACTCCTTAAGTTACTTTAATG |
| tTDH2_Fw_with_tPRM1 | CATTATGCAACGCTTCGGAAAATACGATGTTGAAAATGCGAAAAGCCAATTAGTG |
| tTDH2_Rv_with_VvFLS | GTTTACTGTAAATTGAATAAGATTCCACAATAAATTTAACTCCTTAAGTTACTTTAATG |
| AtFLS_Fw | TTAATCTAATGGCAATTTATTCAATTTTC |
| AtFLS_Rv | ATGGAAGTTGAAAGAGTTCAAG |
| PdFLS_Fw | TTATTGTGGCAATCTATTCAATTTAC |
| PdFLS_Rv | ATGGAATTTGATAGAGTTCAAGC |
| VvFLS_Fw | TTATTGTGGAATCTTATTCAATTTACAG |
| VvFLS_Rv | ATGGATATGGAAAGAGTTCAAG |
| pTPI1_Fw_with_AtFLS | GATGAAGAGATATCTTGAACTCTTTCAACTTCCATTTTTAGTTTATGTATGTGTTTTTTG |
| pTPI1_Rv | GTTTAAAGATTACGGATATTTAAC |
| pTPI1_Fw_with_PdFLS | GATGCGATAGCTTGAACTCTATCAAATTCCATTTTTAGTTTATGTATGTGTTTTTTG |
| pTPI1_Rv | GTTTAAAGATTACGGATATTTAAC |
| pTPI1_Fw_with_VvFLS | GAAAATGCGATAGCTTGAACTCTTTCCATATCCATTTTTAGTTTATGTATGTGTTTTTTG |
| pTPI1_Rv | GTTTAAAGATTACGGATATTTAAC |
| XI-3_HR_DS_Fw_with_pTPI1 | CTAAGTAAGTTAAATATCCGTAATCTTTAAACCCATTTTATTTTTGAGGATTGG |
| XI-3_HR_DS_Rv | GGATAGGACAGGAGCGATAG |
| XI-5_UP_Fw | GAGAAGTCGTTGATAGCATTTCCG |
| XI-5_UP_Rv_with_pCCW12 | GGGCGAAGTGCGCAACCTCGAAGGTTTTCTTTTGCGTGTCGTTCAGCTTAGAGATGTGTC |
| pCCW12_Fw | CGACACGCAAAAGAAAACCTTCG |
| pCCW12_Rv_with_GmFMO | CTGCAGCAATAGTTGCCAAAGCAACAATTAATGGAGACATTATTGATATAGTGTTTAAGC |
| pCCW12_Rv_with_AtFMO | CAGTTGCCAACAAGATTGTCAAAAATAAAGTAGCCATTATTGATATAGTGTTTAAGCG |
| pCCW12_Rv_with_PhFMO | GAAAATAACAGTATACAAAATTAATGACAAAATTTCCATTATTGATATAGTGTTTAAGCG |
| GmFMO_Fw | ATGTCTCCATTAATTGTTGCTTTGGC |
| GmFMO_Rv | TTATGAAGACATTGAGTAAACATGTGG |
| AtFMO_Fw | ATGGCTACTTTATTTTTGACAATC |
| AtFMO_Rv | TTAACCTGAACCCAAACCG |
| PhFMO_Fw | ATGGAAATTTTGTCATTAATTTTG |
| PhFMO_Rv | TTAACCAATGTAAGCTTGTGCTTCC |
| tPRM1_Fw_with_GmFMO | CCAAGATTGGCTCCACATGTTTACTCAATGTCTTCATAAACAGAAGACGGGAGACAC |
| tPRM1_Fw_with_AtFMO | GCACCAAATGTTTACGGTTTGGGTTCAGGTTAAACAGAAGACGGGAGACACTAGC |
| tPRM1_Fw_with_PhFMO | CCAAGACCAAGATTGGAAGCACAAGCTTACATTGGTTAAACAGAAGACGGGAGACACTAG |
| tPRM1_Rv_with_XI-5_Down | GGGCAATTGGGTGTACTATGAAGCAGCCAATACATTTTCAACATCGTATTTTCCGAAGCG |
| XI-5_Down_Fw | GTATTGGCTGCTTCATAGTACACC |
| XI-5_Down_Rv | TGGAGTGTATATGTGAACCTGTAGC |
| X-2_UP_Fw | CGTCTATGAGGAGACTGTTAG |
| X-2_UP_Rv_with_pTDH3 | GATAATGATAAACTCGAACTGAAAAAGCGTGTTTTTTATTCGACCACTTCGAGAGCAAG |
| pTDH3_Fw | GAATAAAAAACACGCTTTTTCAGTTC |
| pTDH3_Rv | TTTGTTTGTTTATGTGTGTTTATTCG |
| UAS_Fw_with_TDH3 | CCAAGAACTTAGTTTCGAATAAACACACATAAACAAACAAATTAGTCAAAAAATTAGCC |
| UAS_Rv_with_AtFLS | GATGAAGAGATATCTTGAACTCTTTCAACTTCCATTTTGTTTGTTTATGTGTGTTTATTCG |
| AtFLS_Fw | TTAATCTAATGGCAATTTATTCAATTTTC |
| AtFLS_Rv | ATGGAAGTTGAAAGAGTTCAAG |
| tTEF1_Fw_with_AtFLS | CATACAGAAAATTGAATAAATTGCCATTAGATTAAGGAGATTGATAAGACTTTTCTAGTTGC |
| tTEF1_Rv | ATAGCGCCGATCAAAGTATTTG |
| X-2_Down_Fw_with_tTEF1 | GTTATTCGATATTGTCGTAACAAATACTTTGATCGGCGCTATCCTGCATAATCGGCCTCAC |
| X-2_Down_Rv | CTCGCCAAGGCATTACCATCC |
| XI-2_UP_Fw | TAACTCTTCGTATGAGGATTTTCG |
| XI-2_UP_Rv_with_pCCW12 | CGAAGTGCGCAACCTCGAAGGTTTTCTTTTGCGTGTCGTTCTATGGCACATTTTTCTG |
| pCCW12_Fw | CGACACGCAAAAGAAAACCTTCG |
| pCCW12_Rv_with_AtFLS | GAAGATGAAGAGATATCTTGAACTCTTTCAACTTCCATTATTGATATAGTGTTTAAGCG |
| AtFLS_Fw | TTAATCTAATGGCAATTTATTCAATTTTC |
| AtFLS_Rv | ATGGAAGTTGAAAGAGTTCAAG |
| tTEF1_Fw_with_AtFLS | CATACAGAAAATTGAATAAATTGCCATTAGATTAAGGAGATTGATAAGACTTTTCTAGTTGC |
| tTEF1_Rv | ATAGCGCCGATCAAAGTATTTG |
| XI-2_Down_Fw_with_tTEF1 | CGATATTGTCGTAACAAATACTTTGATCGGCGCTATCCACAAGTAAAGCTCGTTG |
| XI-2_Down_Rv | ATGGTTGAAAAGGTTACAGAGG |
| XI-2_Verification_UP_Fw | CGTCAATAGCGGCGTCAG |
| XI-2_Verification_Down_rv | CCAAGACCTCTATGGGGTCC |
| X-2_verification_up_fw | GAGTGACAGTGACAACCGC |
| X-2_verification_down_rv | GGTAGGGAGAGTACGCTGTTAC |
| XI-3_verification_up_fw | GTGCTTGATTTGCGTCATTC |
| XI-3_verification_down_rv | CACATTGAGCGAATGAAACG |
| XI-5_verification_up_fw | GAGTCTGGGTCAACCATGATG |
| XI-5_verification_down_rv | GGCTACTGTTCCTAGTAGCACC |

**Table 5.** Constructed DNA modules in this study

| **ID** | **DNA Fragments** |
| --- | --- |
| M1 | XI-3 us-TEF1p-OsF3H-PRM1t-TDH2t-AtFLS-TPI1p- XI-3 ds |
| M2 | XI-3 us-TEF1p-OsF3H-PRM1t-TDH2t-PdFLS-TPI1p- XI-3 ds |
| M3 | XI-3 us-TEF1p-OsF3H-PRM1t-TDH2t-VvFLS-TPI1p- XI-3 ds |
| M4 | XI-3 us-TEF1p-AtF3H-PRM1t-TDH2t-AtFLS-TPI1p- XI-3 ds |
| M5 | XI-3 us-TEF1p-AtF3H-PRM1t-TDH2t-PdFLS-TPI1p- XI-3 ds |
| M6 | XI-3 us-TEF1p-AtF3H-PRM1t-TDH2t-VvFLS-TPIp- XI-3 ds |
| M42 | X-2 us-TDH3-UAS349p -AtFLS-TEF1t- X-2 ds |
| M43 | XI-2 us-CCW12p -AtFLS-TEF1t- XI-2 ds |
| Q1 | XI-5 us-CCW12p -GmFMO-PRM1t- XI-5 ds |
| Q2 | XI-5 us-CCW12p -AtFMO-PRM1t- XI-5 ds |
| Q3 | XI-5 us-CCW12p -PhFMO-PRM1t- XI-5 ds |
| Acc1 | V us-TPIp-ACC1^S659A,S1157A^ -TDH2t-V ds |

**Table 6.** Homology sequences for integration sites at *S. cerevisiae* chromosome.

| **Locus** | **Sequence (5’-3’)** |
| --- | --- |
| X-2 ds | CCTGCATAATCGGCCTCACAGAGGGATCCCGTTACCCATCTATGCTGAAGATTTATCATACTATTCCTCCGCTCGTTTCTTTTTTCAGTGAGGTGTGTCGTGAAAGAAAACCCACAATTAAACTTTCAACAACCGGGCGACTAGGAAGAGAGTAGTGGGCGCGGATGACGAAGGCTAAGGTCACTTCTTCGTTTCCTTTATTGGGGTTTCCGTGTAGCCTTCCCCTGAATAGTGTGGGACGTTTTATGAGAAGCCGTAAGAAATAGGCAAATTGAGTTATGACAAGTAGACATGATGCCGCAGCCTTGCCTGACTTTACGTCTCCTTCATGAATAAGTTTTTCTATCGAGTTCTTTTCCTTTTTTCGCCTTAATTAGCTCAATTAAGCCTGTCCTCACTACTTTTCTTTTTCTTATCGGCTTTGTGCCACACCTAACCTTCGAATGCTGTTTTATTCCGTTCTTACATGGGATGGTAATGCCTTGGCGAG |
| X-2 ds | CCTGCATAATCGGCCTCACAGAGGGATCCCGTTACCCATCTATGCTGAAGATTTATCATACTATTCCTCCGCTCGTTTCTTTTTTCAGTGAGGTGTGTCGTGAAAGAAAACCCACAATTAAACTTTCAACAACCGGGCGACTAGGAAGAGAGTAGTGGGCGCGGATGACGAAGGCTAAGGTCACTTCTTCGTTTCCTTTATTGGGGTTTCCGTGTAGCCTTCCCCTGAATAGTGTGGGACGTTTTATGAGAAGCCGTAAGAAATAGGCAAATTGAGTTATGACAAGTAGACATGATGCCGCAGCCTTGCCTGACTTTACGTCTCCTTCATGAATAAGTTTTTCTATCGAGTTCTTTTCCTTTTTTCGCCTTAATTAGCTCAATTAAGCCTGTCCTCACTACTTTTCTTTTTCTTATCGGCTTTGTGCCACACCTAACCTTCGAATGCTGTTTTATTCCGTTCTTACATGGGATGGTAATGCCTTGGCGAG |
| XI-2 us | TAACTCTTCGTATGAGGATTTTCGATGGAGCAGGATGAGGAGAAATAGTACCACATGTATATATCCATTACAAAAAGGTTTATATACAATTACAATAGACCCTTGTTGGGGTTTCTGAAAAAAGAAGTAGTCGATGCCATCGGCAATAATACGGAATTACGAGAAACACAATCCCGATCCTTTTTTGGGTAATTACTTCACCGATTCTACCGATTTATCATGCCAAAAAAAATTCACCGTGGGTTCTAGAAGTGCCCTTTGAGGATTGTAGCCACTCTAACCCACACGGCCTCCTTACTAGCTGACTAAGGTGACAAAACCGCAAGGACTGGAAAGTCGCCACTCATCTGAAAATTCTCAAGTTTTTCACTACTGAGTTTATGCTTTCGAATTTTTTTGTTCGGTAATAGCACGGCGGTTCGATTCAATTCCGCCGCTCCGAGCGATGCTCCGCAAAACTCAGTAATAAGCTTTCTGATGGTTCACCCCTTTTTTAGCACGCGGGGTGTAACTCAACAGAAAAATGTGCCATAGAA |
| XI-2 ds | CCACAAGTAAAGCTCGTTGACCAGTTGATCAGTTGAGGGGGGTACACACGACTAGCGCTTTCAGATATTAAAAAGTTTAGATGTAGGTTTTAGCGGTAACAGTTATATAAATCGTGTTTCTTCTCTTGATGAAACAAAAAAATGCTAGAAAAACTTTGTCGTTTCTTACTTTTGGTGCGCTTTGCAGTTTTCGTGGCTAGACTTAGAATCATTTCTCCTCAGATTTCTTGATTAAAGTTTGGTGCGAAGCCCTACTCTAACATTGGTGTTCTTCTTTTCATTCACGCAAGTTAAGTCCAGGAAGGTGAGCAAATGCTCATCCTTCTGTTCATGCGTGACGGCTGAATTATCCTTATCTGGCGTACCCGTGCAGCCGTTTCCGTGCCTCGGTTCCTCCGAGATATCCTTAGGGACCGCCAGGGACCATGATTGCGTCAACTGTTGTCACCGCTCCAGAGGATCCTCTGTAACCTTTTCAACCAT |
| XI-3 us | AGTTACTTGCTCTATGCGTTTGCGCATCCTCTTTTTACTTTTTTTTTTTCAGTAAAGCCTAAGCATAAATCGTTTTATACGTACGACACGTCAACTTTTCTTGGTTAGTAGTGGCAATCTCTGCAATACATACAGGGAGTCATGGTCTATCATCTTGTCCAATCAAAGAAGCATCGGTTCAGATCGAGCAAACTGTAGGGAGAAAGGAAAGTAGAAATGCAGAGTGTGCTATATGTCCAATCTCGGTTTTGTAGTTTGGATGTCATTAAGATCTACCACCCAACCGGCTGCTTTCATGTGGAACAGAAAAGAAATCGGGGCGCTTCCTCTTCTGTATTCCTTTAATTAACGTTTTTATTCAGCCATCTAACCATCATACCCCCATACGGTAACAAAACCTCTTCTAAGAAAAGAAGTCTCTGCTCCTCCGCCATCTTATTTTTATTCGCTGCGCGCGTTTATTGTCGCATCGCTAGCCAGCAAAAAGTTGGTTGCCTTTTTTTACCTAAAAAAGACACATCTAACTGATTAGTTTTCCGTTTTAGGATATTGACGCCAAGCGTGCGTCTGATT |
| XI-3 ds | TTACGTGGATTGAGCCAGCAATACAGATCATTATTAAACTGTTTTGTACATGATGTTAGTATATAATCGTAAAGCTTTTCTAATATGTATACCTTATACATGGAACTCCACAGAACTTGCAAACATACCAAAAATCCTTTATTCTTGTTCACTCATTTTACATCAAAAAATAATATTTCAGTTATTAAGGAAAATAAAAAAATAGATTAGAGAAGCATTTTGAAGAAATAGTATATTCTTTTATTGAACCTAAGAGCGTGATATTTTTACTCGAAATAAAATACGAAAAATCTATACACTCATCTTTCCGACTACTATTGGCTCCTGCTCAAAAAAAGAGGGAAAAAAAGCTCCAAAATTCTATCTTTTCCTATCGCTCCTGTCCTATCCTTATTACGTTCATTACTATTTTAATACTATCCATTCTTTTATTTTCAGTCTAAAAAAAACATTTCTCATAACGGGAAAAGCAAAAAAATGTCAAGCTTATACATCAAAACACCACTGCATGCATTATCTGCTGGTCCGGATTCTCA |
| XI-5 us | GCGGAGAAGTCGTTGATAGCATTTCCGAAGGCTTTTCCACATTGACTGCAGTATCTAAAAGAGCTCCAGCAGCGGTCTTAGTAGTTCCTGTGCTTGCCGCTTTGCAGTATACAGGGACATTGGGAACTACGGAGGAAAAATCGGAATGCTCTGAGCTCTTCTCAAAACCAGACGAACTCGTCTTATTTCTCAGTAAATTACCCAACATATAAACTCTCCCTCTTCACCTTCTCTACAAGGCCACACACAAGACAGTTTTAACAGGCAAATGGTACTGGAGGCTCTCACCAATGGGGTTTAAGACGCTCCAAACACACGCTCTCCTTTTTTTCTTTATATATCGGTAGCAACGAAAGCTAGTCGCAGAACGTTACACACTAAAGCAGATCAATCAATCAATCCTGAGAACCTGCTTCGTTCAAACCATCCACTAGAAGAAACACGCACGTGTACCCAAAAATAGAGACAAACAAACAGACAAAAATAACAATGATTCCTACCGACTAAAGTAAAACAACACTCACGTAATAAAAAATCCTAGATACAACTCACTTGTATTCCTCTCTTCTTTGACCATCACTAAAGCTTCTCTCTTACTATATTCCTCAAATAAAAAAAAGTTGCAATGATTACTTACCAATGTGCCATAAACTCCGTGCACCA |
| XI-5 ds | TTGTAAAACAGGTATTGGCTGCTTCATAGTACACCCAATTGCCCACAACCCAAGTTAATCATAAGCTCTTGTTGTCGATGAATTGCTCAAAATGTGGCCATTCTTTCAATTTTGCACCTCTAAAACTGCTCCGTGGGTTGTGACAACAACCTTAAGGGTAATGGACTTTTTTAGAAAGACAAATGAGATGATTTCACCTACTGGTATATCATTTATTTATACACGACCGTTTTGACATTTCAGAGTTGCACGTTGGTATATCCTAATATTCTCACAAGCATCACAGTGAAGAAACGCAAGAAAATGGAAAAAATTCCCAGGTGGTTGTTGTTTTCTCTTATATCGTCGGTGCTGTGCATACTTGGGGCCCTGTGTGTGCCGTTGTTATCGGTTGCCTTCGATAGCAAGCGCAATAGCCAATCTAAGTTGGTCAACTATGGTCTTTCTCTAAGTGCCGGATCTATGATC |
| V us | GAATGTCCGGTAATTCCGTTTGCTATATTCTATTTGTTGATACTGATGAGCATCAAAAAATTATGTAATAAGGGTATCAATTCGCATTCTTATATTTAATACATACCCAGTTGTTTGTAGCTGGTTCATATTTAGCGGCAATTCTCTGTTGCGTAAATGAAAATATTAATGTAAACAAAAAAAGACCAAAACATTTTAGCAGTGTAAGAAGGTGTACTGATACAAAATGTGTTTAGAGTCTACTGATATGTTACTGACCGTTCGTTGGGAAAAAAATACTGTATCATTTATTAATCAAAAGCGACTTTTGGTGGAATATTATGATATGTGTTGTTAAAATATGACGTAATTTTAGAATTGTCTGATTCGTATTCAAATTTGGTGAAGGAATAACGCAGAGTTGACAATTTAATAGAATGGATTAATCGTAATTTTCAGAAACGTAGAAAAAGAAAAACAATTAAAACATTATATTAAGATTATTGATTTGCCTTTTAAGGGTCCATACTCCT |
| V ds | ATATATATACCTGAGAAACTGGCGAGTAATTTGATAATTGTTCGGATTGCATTGTTGCCGAATCCTATAACATTAGGTATACATAGTATACAAGCAATTCTCCCGACGTTATAGGAATTCACAAAAGAGAATCGATGGTGTTACTTTATCTTCCTTCCCGTTATACTTTGTCTTTCATTATCCTATTAGATTACATTTCAGTTTCCATCAATTTTTGATGGCTGTCTCTCAATTTATATTATCTTCTTATACCGTATATGATGGTATACTAGTTTACAATATTAGACGATGGATGATGATTGGTTCTTATCATAATTTGATTTCGGCAGAAGCAATATTAGAGGTATTGTTGTAACGAAATTCCAATGTCATCTGCTTAGTATTATTAATGTTACCTGAATATTATCACATGCTGCTTAAAAATGTGTTATAAGTATTAAAATCTAGTGAAAGTTGAAATGTAATCTAATAGGATAATGAAACATATGAAACGGAATGAGG |
